# Gut microbiomics reveals host–microbe crosstalk during sexual differentiation in the shortfin eel *Anguilla bicolor*

**DOI:** 10.1093/ismeco/ycag241

**Published:** 2026-08-22

**Authors:** Guo-Jie Brandon-Mong, Tzong-Huei Lee, Yi-Lung Chen, Tsun-Hsien Hsiao, Guan-Lun Wu, Yi-Li Lai, Chieh-Yin Weng, Ronnie G Gicana, Yu-Ting Yen, Yi-Fan Xu, Tzi-Yuan Wang, Yin-Ru Chiang

**Affiliations:** Biodiversity Research Center, Academia Sinica, Taipei 115, Taiwan; Institute of Fisheries Science, National Taiwan University, Taipei 106, Taiwan; Department of Agricultural Chemistry, National Taiwan University, Taipei 106, Taiwan; School of Medicine, National Tsing Hua University, Hsinchu 300, Taiwan; Institute of Molecular and Cellular Biology, National Tsing Hua University, Hsinchu 300, Taiwan; Institute of Fisheries Science, National Taiwan University, Taipei 106, Taiwan; Biodiversity Research Center, Academia Sinica, Taipei 115, Taiwan; Biodiversity Research Center, Academia Sinica, Taipei 115, Taiwan; Biodiversity Research Center, Academia Sinica, Taipei 115, Taiwan; Institute of Bioinformatics and Structural Biology, National Tsing Hua University, Hsinchu 300, Taiwan; Biodiversity Research Center, Academia Sinica, Taipei 115, Taiwan; Biodiversity Research Center, Academia Sinica, Taipei 115, Taiwan; Biodiversity Research Center, Academia Sinica, Taipei 115, Taiwan; Department of Agricultural Chemistry, National Taiwan University, Taipei 106, Taiwan

**Keywords:** *Anguilla*, *Deinococcus*, gut microbiota, host–microbe interactions, sexual differentiation, sex hormones, steroid metabolism

## Abstract

The global decline in anguillid eel populations has intensified interest in understanding their biology for conservation and aquaculture. While host–gut microbiota interactions are well-characterized in homeotherms, these relationships remain poorly understood in poikilotherms during sexual differentiation. We examined gut microbiota dynamics across developmental stages in the shortfin eel *Anguilla bicolor*, which exhibits early sexual differentiation and a relatively short life cycle. Glass eels were cultivated in controlled freshwater conditions for 3 years, with sampling at key stages: glass eel, elver, sex-undetermined eel, and sex-determined eel. Full-length 16S rRNA gene sequencing revealed significant compositional shifts during development, with higher bacterial richness in adults versus younger eels. Early stages were dominated by Pseudomonadota, while sex-determined adults showed increased Deinococcota abundance. Network analysis identified *Deinococcus, Sphingomonas*, and *Variovorax* as key genera in sex-determined eels, with positive correlations between anti-Müllerian hormone gene expression and these taxa. We collected 66 gut bacterial strains capable of metabolizing sex hormones under microaerobic conditions. These isolates, representing 22 genera across four phyla, demonstrated diverse metabolic capabilities from partial oxidation to complete steroid mineralization. Multiple strains achieved complete estradiol degradation as single isolates—a rare metabolic capability of environmental microorganisms. Comparative genomic analysis revealed widespread steroid-metabolizing genes, with *Deinococcus* species showing previously unreported hormone degradation capabilities. Our microbiomics analysis demonstrates that gut microbiota composition and function are intimately linked to eel sexual development, suggesting bidirectional host–microbe interactions influencing reproductive physiology. These findings advance understanding of host–microbiota interactions in aquatic vertebrates and provide implications for eel aquaculture and conservation.

## Introduction

**Figure 1:**
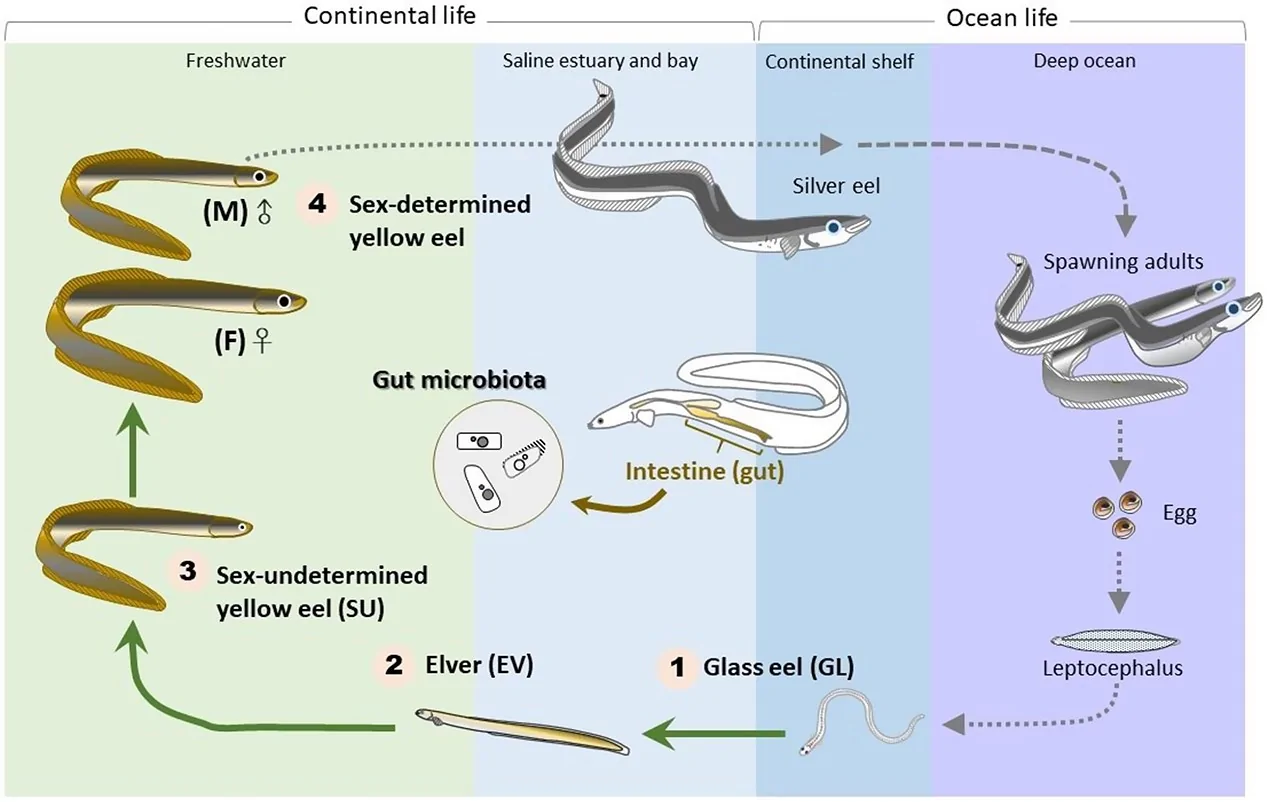
The complete life cycle of *Anguilla* spp. The numbers represent eel collected at different development stages: (1) GL, glass eel stage; (2) EV, elver stage; (3) SU, sex-undetermined juveniles; and (4) M and F, sex-determined male and female adults.

While most anguillids are marine species, the genus *Anguilla* (eels; Fig. 1) is unique in performing long-distance migrations between fresh and marine waters. *Anguilla* populations have declined dramatically worldwide due to high market demand and overfishing [1–3]. For example, the European eel (*Anguilla anguilla*) has experienced a population decline exceeding 90% since the 1980s and has been classified as critically endangered on the International Union for Conservation of Nature (IUCN) red list since 2008 [4, 5]. Similarly, the Japanese eel (*Anguilla japonica*), a temperate Asian species, is listed as endangered, with its population decreasing by more than 50% over three decades [5].

Unlike the extensively studied Japanese eel, the ecology and physiology of the tropical Asian shortfin eel (*Anguilla bicolor*) remain poorly understood. Currently listed as near threatened [5], *A. bicolor* exhibits distinct life history traits compared to *A. japonica*. In general, *A. bicolor* undergoes sexual differentiation earlier than *A. japonica*. Tropical *A. bicolor* completes sexual development within approximately 4 years [6, 7], while in *A. japonica*, gonadal sex differentiation may initiate by 3–4 years, with some individuals remaining undifferentiated for up to 7 years [8]. Additionally, sexually differentiated *A. bicolor* are frequently encountered in freshwater environments [9]. Beyond their extraordinary migratory ecology, eels exhibit remarkably complex life cycles and sexual development patterns. Their reproductive biology is governed by sophisticated hormonal mechanisms that have been most extensively studied in Japanese eels. This research has revealed pronounced sexual dimorphism, with females typically achieving enhanced growth rates and larger body sizes compared to males [10]. These morphological differences are complemented by distinct sex-specific molecular markers: males demonstrate elevated expression of the gonadal anti-Müllerian hormone (*amh*) gene [11], while females exhibit higher expression levels of gonadal vitellogenin receptor (*vtgr*), which encodes an oocyte membrane-specific receptor essential for egg development [12].

The primary sex steroids in eels, particularly Japanese eels, include two androgens—testosterone and 11-ketotestosterone—and one estrogen, 17*β*-estradiol (hereafter referred to as estradiol) [13]. Testosterone serves as a principal androgen and as a precursor for other sex steroids, including 11-ketotestosterone and estradiol [14]. 11-Ketotestosterone is critical for testicular development and spermatogenesis, particularly during the final stages of sperm maturation [15]. Similar to mammals, estradiol serves as the primary female sex hormone in eels. Produced in the ovaries through aromatization of testosterone, this female-specific steroid is essential for ovarian development and vitellogenesis (yolk formation in eggs) [14, 16]. It stimulates the liver to produce vitellogenin, a protein crucial for egg development. Using Japanese eel as a model organism, researchers have observed that concentrations of these sex steroids increase significantly during sexual maturation, regulating the silvering process—the metamorphosis from yellow to silver eels preceding their oceanic spawning migration [17–20]. The balance between these hormones shifts dramatically throughout the eel’s unique life cycle, particularly during the silvering phase (transition from yellow to silver eel). This metamorphic transformation is characterized by significant digestive tract regression, with 11-ketotestosterone playing a key role in inducing gut degeneration alongside other silvering-related changes such as eye enlargement and gonadal development [19]. The progressive gut atrophy serves as an adaptive preparation for the extensive nonfeeding migration, during which silver eels must survive solely on accumulated energy stores during their oceanic spawning journey.

Like all complex organisms, eels exist not as isolated biological entities but as holobionts—integrated systems of host and associated microbial communities [21]. This host–microbiota framework offers a promising lens for understanding sexual differentiation and reproductive physiology in anguillid eels. Accumulating evidence demonstrates that gut microbiota significantly influence critical host functions, particularly reproduction and endocrine regulation [22]. Notably, recent studies have shown that gut microbiota can actively modulate host hormone profiles through enterohepatic circulation, creating bidirectional communication between microbial communities and the host endocrine system [23, 24]. This regulatory capacity extends to fish sex development, where gut bacteria have been shown to play important roles [25–27].

Despite these advances, the relationship between host sex hormones and gut microbiota composition remains poorly understood in aquatic animals, particularly in anguillid eels. Current research on eel gut microbiota is limited to only six studies spanning different species and life stages: juvenile of *A. anguilla* [28], juvenile through silver adult of *A. anguilla* [29], juvenile of *A. australis* [30], glass eel of *A. rostrata* [31], immature and adult of *A. marmorata* [32], and sex-determined adult of *A. japonica* [33]. Critically, research on *A. bicolor* gut microbiota beyond the juvenile (i.e. elver) stage remains absent [30]. Further complicating our understanding, previous studies were conducted across varied environmental conditions—with factors such as pollutants, habitat conditions, salinity, and temperature all capable of influencing gut microbiota composition. This environmental heterogeneity has left many fundamental aspects of eel–microbiota interactions unexplored. Controlled environment studies could therefore provide crucial insights into the specific bacterial taxa and their functional roles throughout eel development.

Here, we hypothesize that host–gut microbiota interactions drive developmental programming and sexual differentiation in the shortfin eel *Anguilla bicolor*. We aimed to: (i) characterize the correlations between sex hormones and gut microbiota across different life stages, and (ii) identify key gut microbes capable of hormone metabolism in sex-determined *A. bicolor* eels. To minimize confounding environmental factors that could affect wild populations, we sourced *A. bicolor* specimens from a controlled aquaculture facility. Our microbiomics approach demonstrates that host–gut microbiota interactions play a crucial role in developmental programming and sexual differentiation of *A. bicolor*, providing valuable insights for both eel ecophysiology and sustainable aquaculture practices.

## Materials and methods

Eels (*A. bicolor*) were collected from a fish farm in Hsinchu, Taiwan. Details of eel husbandry—including the culture system, nutritional composition of bloodworms and feed pellets, sample collection, and animal-experiment ethics—are described in the Supplementary Materials and Methods. For each specimen, body length, body weight, gonadosomatic index (GSI), sex hormone levels, and gene expression were measured. For culture-independent microbiome analysis, full-length 16S rRNA genes from gut microbes were sequenced using PacBio HiFi technology, quality-filtered, and taxonomically classified. Bioinformatics analyses included alpha and beta diversity calculations, linear discriminant analysis (LDA), Venn diagram comparisons, and co-occurrence network analyses. For culture-dependent analysis, gut bacteria were isolated using either rich medium or mineral medium, each supplemented with 1 mM of individual sex steroids (testosterone, 11-ketotestosterone, or estradiol). Functional validation involved identifying microbial steroid metabolites through thin-layer chromatography. Functional genomic analysis was performed on NCBI RefSeq genomes sharing ≥ 98.7% 16S rRNA gene identity with the eel gut bacterial isolates and commercially obtained *Deinococcus* strains. Detailed protocols are provided in SI Supplementary Materials and Methods.

## Results

### Morphometric and physiological characterization of developmental stages in captive *Anguilla bicolor*

**Figure 2:**
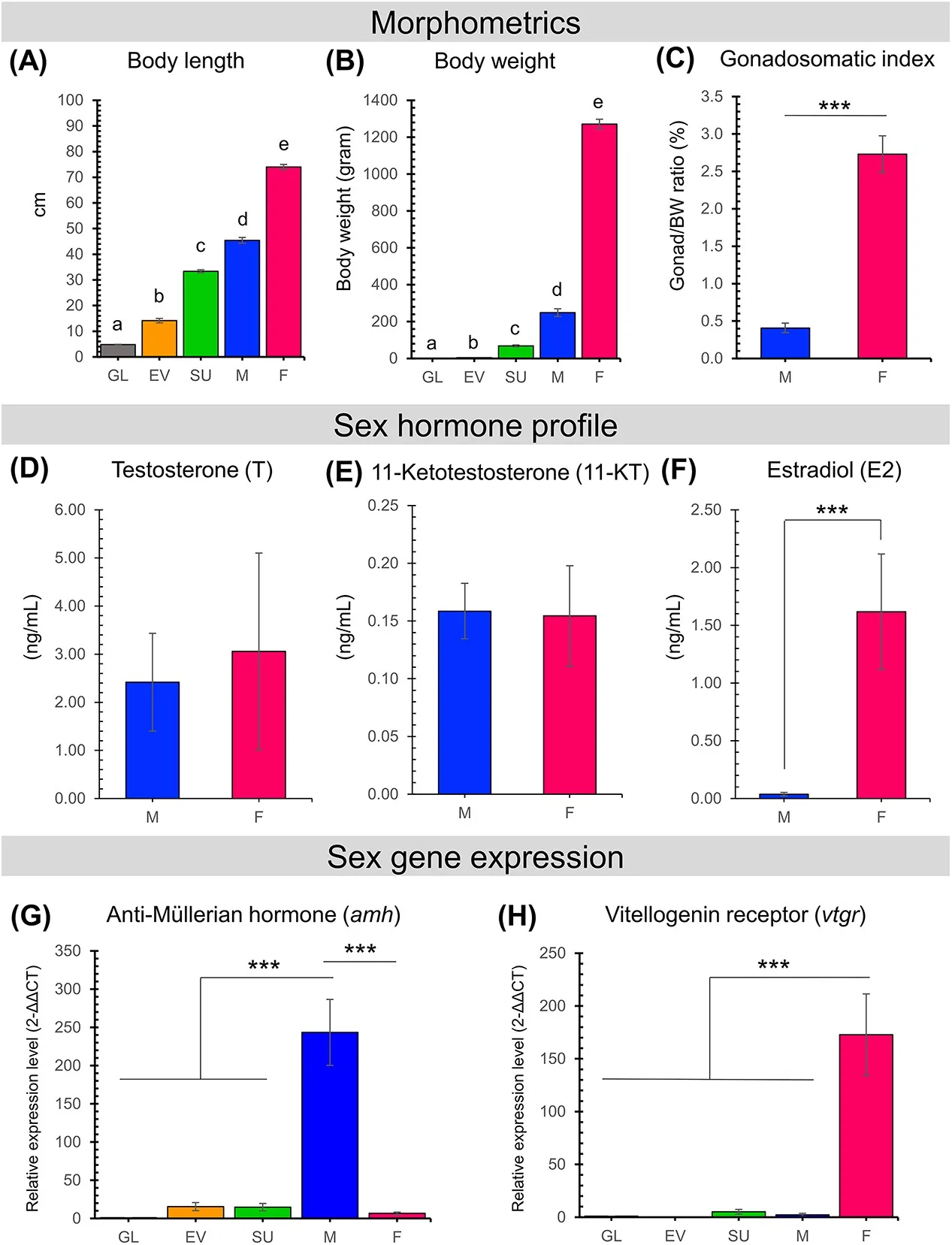
The morphometrics, sex hormone profiles, and expression of the selected sex-specific genes (*amh* and *vtgr* for male and female, respectively) of eel individuals collected at different development stages. The eel morphometrics determined in this present study include (A) body length (cm), (B) body weight (gram), and (C) Gonadosomatic index (GSI; %). Major sex hormones detected in the sex-determined eels include (D) testosterone, (E) 11-ketotestosterone, and (F) estradiol. Sex-specific genes studied in the eel samples include (G) *amh* and (H) *vtgr*. Number of eel individuals collected at different development stages: GL, *n* = 6; EV, *n* = 8; SU, *n* = 10; M, *n* = 10, and F, *n* = 10. Different letters (a ~ e shown in panels A and B) indicate significant differences between groups (*P* < .01). Data with normal distributions were analyzed using Welch’s *t-*test; data with non-normal distributions were analyzed using the Wilcoxon rank-sum test; ^***^P < .001.

Wild glass eels were collected from an estuary in Hualien (eastern Taiwan) and identified as *A. bicolor* through maximum likelihood phylogenetic analysis of *COI* sequences (Supplementary Fig. S1). The bloodworms used to feed the glass eels were identified as *Chironomus circumdatus* (Supplementary Fig. S2). Cholesterol was detected in both bloodworm and feed pellet samples, whereas androgens (testosterone, dihydrotestosterone, androstenedione, and androstadienedione) and estrogens (estrone, estradiol, and estriol) were below the detection limit of UPLC–APCI–HRMS (Supplementary Fig. S3A). Cholesterol content was 0.19 ± 0.03 mg/g wet weight in bloodworms and 10.55 ± 0.24 mg/g wet weight in feed pellets (Supplementary Fig. S3B). The collected glass eels were cultivated in a freshwater fish farm for 3 years, with individuals sampled (*n* ≥ 6 per stage) at key developmental milestones: glass eel (GL), elver (EV), sex-undetermined eels (SU), and sex-determined eels (male [M] and female [F]). Morphometric analysis demonstrated progressive increases in body dimensions throughout development. Body length increased significantly across all developmental stages (all pairwise comparisons *P* < .01), progressing from 4.8 ± 0.12 cm in GL to 14.2 ± 0.86 cm in elvers, 33.4 ± 0.59 cm in SU, 45.4 ± 1.14 cm in M, and 74.0 ± 1.01 cm in F (Fig. 2A). Body weight exhibited a parallel developmental trajectory: GL (0.1 ± 0.01 g), EV (4.6 ± 0.84 g), SU (68.4 ± 5.07 g), M (248.7 ± 20.98 g), and F (1271.2 ± 26.61 g), with all groups significantly different from one another (*P* < .01) (Fig. 2B). Sexual dimorphism was evident in the GSI, with females displaying a substantially higher index (2.7% ± 0.24%) compared to males (0.4% ± 0.06%, *P* < .001) (Fig. 2C).

Sex determination in adult eels was confirmed through integrated morphological assessment, genetic marker analysis, and hormonal profiling. Steroid hormone analysis revealed distinct profiles between sexes, with testosterone serving as the predominant sex steroid in both sex-determined males and females (mean serum concentrations > 2.0 ng/ml) (Fig. 2D). Conversely, 11-ketotestosterone remained consistently low (<0.2 ng/ml) across both sexes (Fig. 2E). ELISA quantification revealed pronounced sex-specific differences in estradiol concentrations. Females exhibited markedly elevated estradiol levels (1.62 ± 0.50 ng/ml) compared to males (0.04 ± 0.02 ng/ml, *P* < .001) (Fig. 2F). Despite this difference, testosterone concentrations remained comparable between sexes, with males showing 2.4 ± 1.02 ng/ml and females 3.1 ± 2.05 ng/ml (Fig. 2D).

To further validate sex determination, reverse-transcription quantitative polymerase chain reaction analysis was performed targeting sex-specific molecular markers: anti-müllerian hormone (*amh*) as a male-specific marker and vitellogenin receptor (*vtgr*) as a female-specific marker. Gene expression patterns provided clear discrimination between sexes and developmental stages. M demonstrated markedly elevated *amh* expression (243.4 ± 43.16) compared to all other developmental groups: GL (1.0 ± 0.01), EV (15.5 ± 0.84), SU (14.7 ± 4.88), and F (6.7 ± 1.55, *P* < .001) (Fig. 2G). In contrast, *vtgr* expression exhibited a distinct developmental progression, increasing from GL (1.0 ± 0.00) through EV (0.1 ± 0.08) and SU (5.2 ± 2.23), ultimately reaching peak expression in F (172.8 ± 38.69). M maintained consistently low *vtgr* expression (2.2 ± 1.50, *P* < .001) (Fig. 2H). This comprehensive analysis integrating morphometric data, gene expression profiles, and sex steroid concentrations establishes *amh* and *vtgr* as robust and reliable molecular markers for sex determination in male and female eels, respectively.

### Dynamic changes in gut microbial diversity of *Anguilla bicolor*

**Figure 3:**
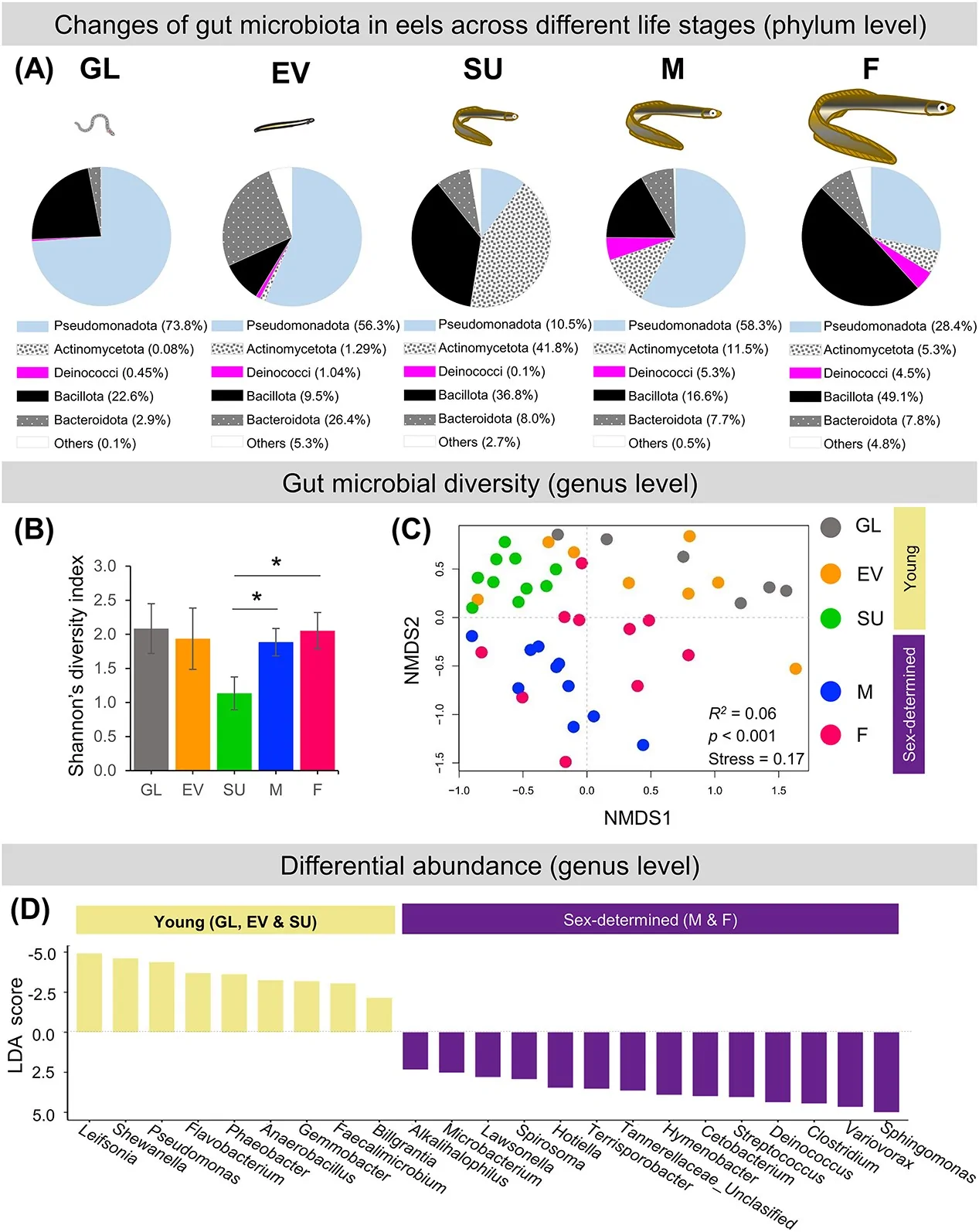
Changes of gut microbiota in eels across different life stages. (A) Pie charts show the dominant microbial phyla in the eel gut; (B) Shannon’s diversity index of the eel gut microbiota (genus level). Data with normal distributions were analyzed using a Welch’s *t-*test; ^*^P < .05; (C) gut microbial composition analysis (genus level) based on Bray–Curtis dissimilarity and visualized in NMDS (PERMANOVA: *R*^2^ = 0.09; *P* < .001); (D) differential abundance of gut microbial genera (LEfSe). Barplot shows significantly dominant bacterial genera in either young (GL, EV, and SU; *n* = 24) or sex-determined adults (M and F; *n* = 20) groups. Genera are ranked by their LDA score.

Gut microbiota composition was characterized across eel developmental stages using 16S rRNA gene amplicon sequencing (Fig. 3). The biomass of microbiota in gut samples from GL and freshwater samples was low, yielding a minimum of 2933 clean reads. Rarefaction analysis demonstrated that most eel samples reached saturation at approximately 600 reads, while feed pellet samples—the primary food source for elvers and adult eels—approached plateaus at the full 2499 reads (Supplementary Fig. S4). Therefore, bacterial 16S rRNA gene sequences were normalized to 2499 reads per sample. Shannon diversity index curves plateaued at 600 reads across all sample types, including environmental controls, confirming adequate sequencing depth.

Phylum-level analysis revealed dramatic microbial community restructuring throughout eel development (Fig. 3A). GL microbiomes were dominated by Pseudomonadota (73.8%), with Bacillota (22.6%) and Bacteroidota (2.9%) as minor constituents. During the EV stage, Pseudomonadota remained predominant (56.3%) but showed reduced abundance, coinciding with increased Bacteroidota representation (26.4%) and emergence of Spirochaetota (4.2%).

SU exhibited the most pronounced taxonomic shift, characterized by Actinomycetota (41.8%) and Bacillota (36.8%) co-dominance, representing a departure from the Pseudomonadota-dominated profile of earlier stages. Adult stages showed sex-specific patterns: M demonstrated renewed Pseudomonadota dominance (58.3%), while F displayed peak Bacillota abundance (49.1%). Notably, Deinococcota showed progressive enrichment from juvenile to adult stages, contrasting with the early-stage dominance of Pseudomonadota (Fig. 3A).

Alpha diversity, measured by Shannon's index at the genus level, exhibited stage-specific fluctuations: GL (2.1 ± 0.365), EV (1.9 ± 0.450), SU (1.1 ± 0.240), M (1.9 ± 0.200), and F (2.1 ± 0.267) (Fig. 3B). Simpson’s index showed a similar trend to that of Shannon’s index (Supplementary Fig. S5). The marked reduction in diversity during the SU stage suggests a bottleneck effect during sexual differentiation. Beta diversity analysis using nonmetric multidimensional scaling (NMDS) based on Bray–Curtis dissimilarity revealed distinct community structures across developmental stages. NMDS ordination demonstrated a good fit (stress = 0.12), and PERMANOVA confirmed significant differences among life stages (*R*^2^ = 0.06, *P* < .001) (Fig. 3C). Integration of environmental controls demonstrated that eel gut communities formed discrete clusters, clearly separated from surrounding freshwater, red worms (primary food source for GL), and feed pellets (PERMANOVA: *R*^2^ = 0.06, *P* < .001; Supplementary Fig. S6). Pairwise comparisons between consecutive developmental stages confirmed significant community transitions: GL-EV (*R*^2^ = 0.23, *P* < .001), EV-SU (*R*^2^ = 0.18, *P* < .001), SU-M (*R*^2^ = 0.24, *P* < .001), SU-F (*R*^2^ = 0.12, *P* = .006), and M-F (*R*^2^ = 0.12, *P* = .017), indicating continuous microbiome restructuring throughout development (Supplementary Fig. S7).

Linear discriminant analysis effect size (LEfSe) identified distinctive bacterial genera distinguishing sex-determined eels (M and F) from younger eels (GL, EV, and SU) (Fig. 3D). Sex-determined eels harbored 14 significantly enriched bacterial genera, with *Sphingomonas* showing the highest discriminatory power (LDA score: −4.99). Other notably enriched adult-associated genera included *Variovorax* (−4.66), *Clostridium* (−4.44), *Deinococcus* (−4.37), and *Streptococcus* (−4.04).

Conversely, younger stages were characterized by nine significantly enriched genera (*P* < .05; LDA score > 2). *Leifsonia* exhibited the strongest juvenile association (LDA score: 4.92), followed by *Shewanella* (4.62), *Pseudomonas* (4.38), and *Flavobacterium* (3.70). Further analysis revealed sex-specific microbial signatures in sex-determined eels (Supplementary Fig. S8). Males showed significant enrichment of six bacterial species (*P* < .05; LDA score > 2), including *Sphingomonas glacialis* (LDA score: 5.23), *Variovorax rhizosphaerae* (4.84), and *Deinococcus aquiradiocola* (4.28). Females exhibited enrichment of three species: *Staphylococcus warneri* (−4.20), *Deinococcus geothermalis* (−4.17), and *Anoxybacillus kestanbolensis* (−3.36).

**Figure 4:**
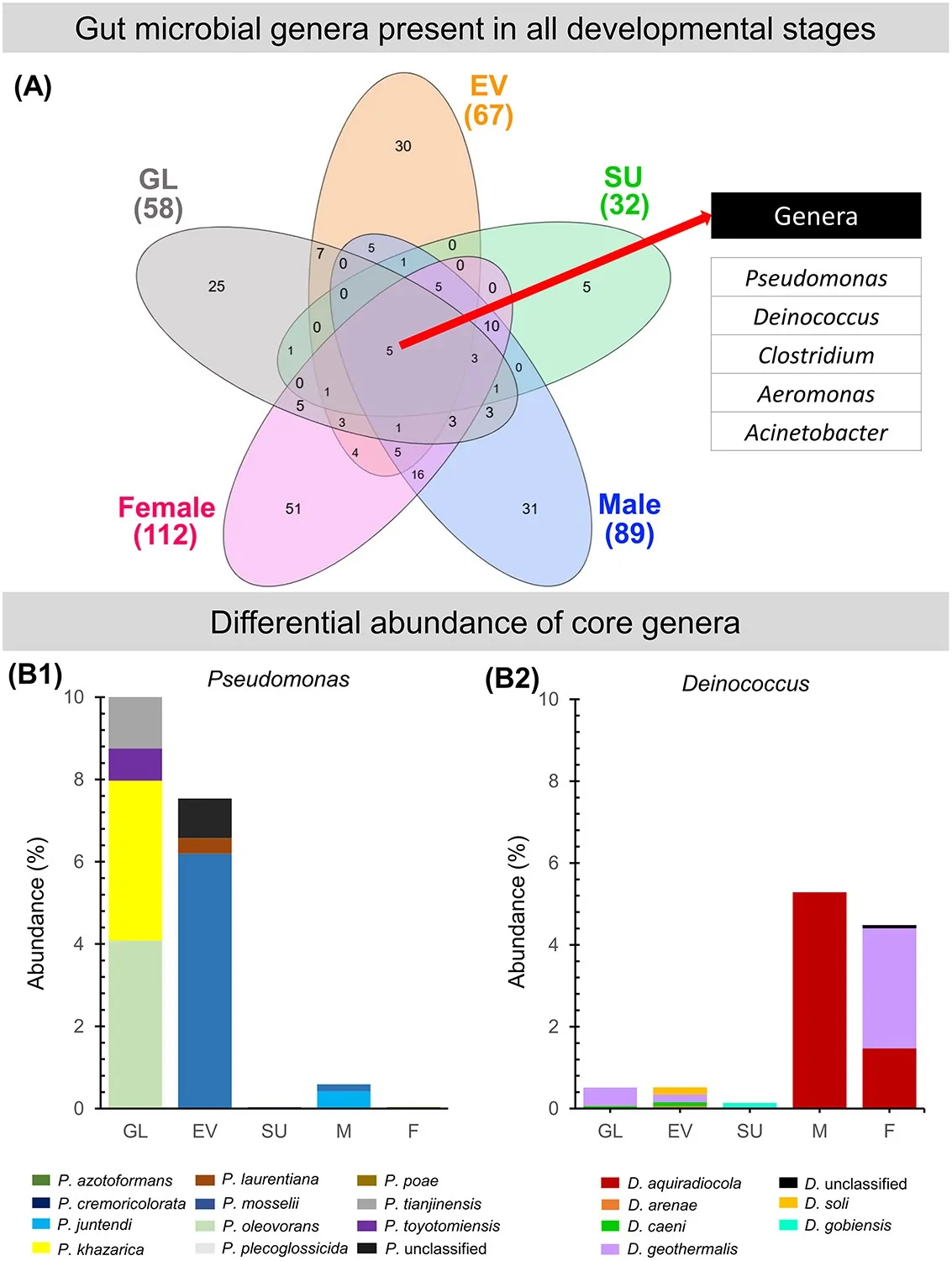
(A) Venn diagram analysis of gut microbiota identified five genera (*Pseudomonas, Deinococcus, Clostridium, Aeromonas*, and *Acinetobacter*) persistently present across all developmental stages, with sex-determined adults showing higher bacterial richness (number of genera) than younger eels. (B) Relative abundance changes of two representative core genera across eel development. (B1) *Pseudomonas* demonstrated a progressive decline in relative abundance from glass eel to adult stages. (B2) *Deinococcus* showed an increasing abundance trend throughout eel development from glass eel to adult stages.

Venn diagram analysis revealed no core genus or species (prevalence > 80%) shared across all developmental stages in either unrarefied or rarefied count tables (Supplementary Figs S9 and S10), with core taxa identified within each stage listed accordingly. Nevertheless, five bacterial genera—*Deinococcus, Pseudomonas, Acinetobacter, Clostridium*, and *Aeromonas*—were persistently present across all developmental stages (Fig. 4A). Among these taxa, *Pseudomonas* demonstrated a progressive decline in relative abundance from GL to sex-determined eels (Fig. 4B1), while *Deinococcus* exhibited the opposite trajectory, increasing throughout development (Fig. 4B2). At the species level, *Aeromonas hydrophila* and *Clostridium*_Unclassified were shared across all eel developmental stages (Supplementary Fig. S11). Bacterial richness (number of genera) was highest in F (112 unique genera), followed by M (89), EV (67), GL (58), and SU (32) (Fig. 4A). This pattern of bacterial richness was consistent across both taxonomic levels, as similarly observed at the species level (Supplementary Fig. S11). These patterns suggest increasing microbiome complexity and specialization with sexual differentiation and adult development.

### Microbial co-occurrence networks across eel life stages

**Figure 5:**
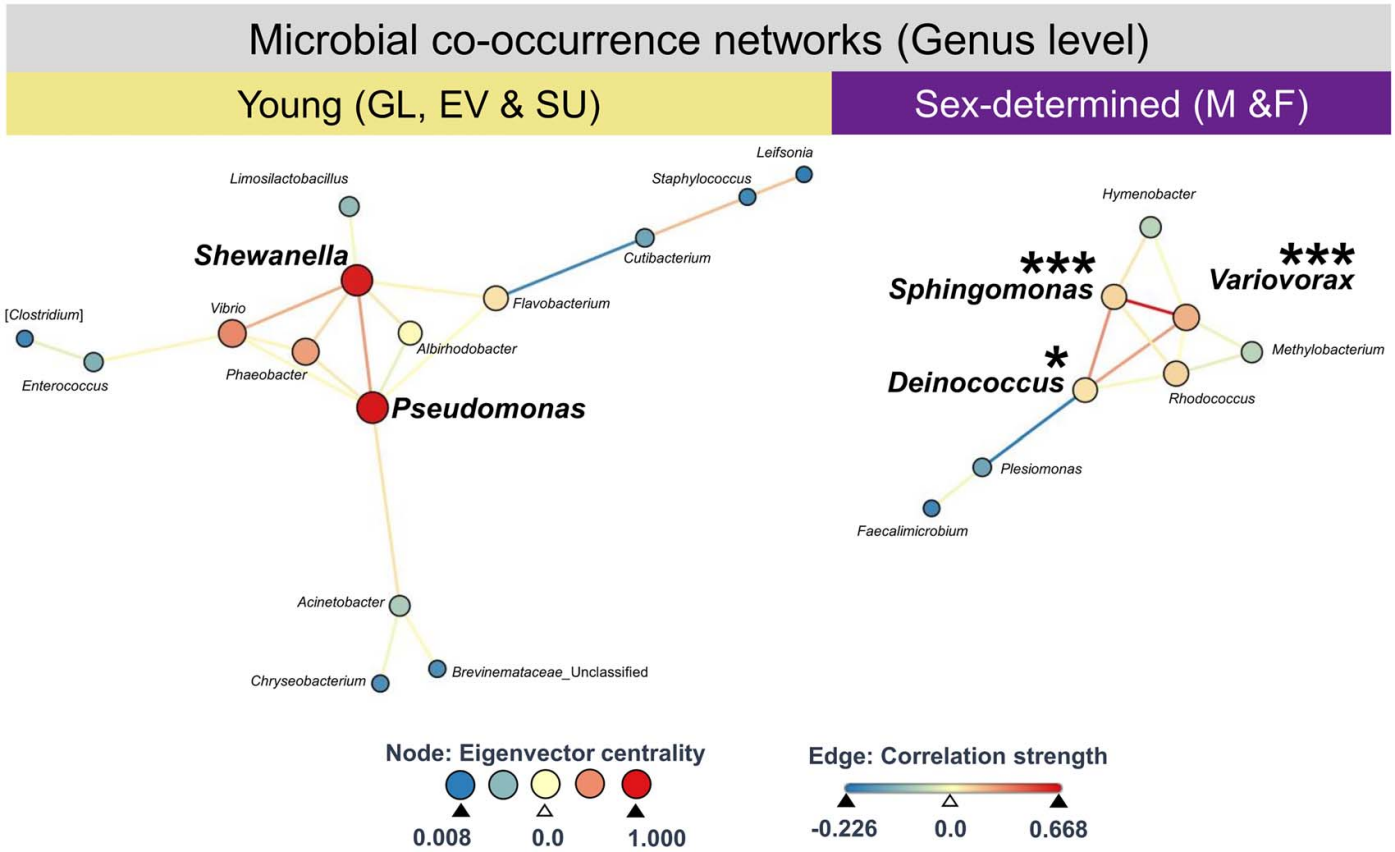
Microbial co-occurrence subnetworks at the genus level across eel life stages and correlations between microbial genera and sex-specific gene expression. Each node represents a microbial genus, with node color and size indicating the eigenvector centrality score. Edge color represents the correlation strength between nodes, reflecting associations between microbial genera. Correlations were calculated using FastSpar with 20 iterations and 10 000 bootstrap replicates for *P*-value estimation. Asterisks indicate significance levels for correlations between microbial genera and the expression of *amh*: ^*^P < .05; ^**^P < .01; ^***^P < .001. Detailed correlation strengths are available in Supplementary Table S1.

The microbial co-occurrence networks at the genus level across eel life stages (GL, EV, SU, M, and F) are presented in Fig. 5. Network analysis revealed two distinct subnetworks corresponding to early (GL, EV, and SU) and mature (M, F) life stages, each dominated by a different central genus. The early life-stage subnetwork consisted primarily of six genera of the phylum Pseudomonadota—*Albirhodobacter, Flavobacterium, Phaeobacter, Pseudomonas, Shewanella*, and *Vibrio*. Centrality metrics identified the bacterial genus *Pseudomonas* as the most influential node (Rank 1: eigenvector = 1.000, degree = 6, closeness = 0.539, betweenness = 0.068), followed by *Shewanella* (Rank 2: eigenvector = 0.984, degree = 6.000, closeness = 0.500, betweenness = 0.038), *Vibrio* (Rank 3) (Fig. 5, left panel; Supplementary Table S1). In contrast, the mature-stage subnetwork was centered on *Variovorax*, which emerged as the most influential genus in this network (eigenvector = 0.669, degree = 5.000, closeness = 0.700, betweenness = 0.008; Fig. 5, right panel; Supplementary Table S1). *Variovorax* formed significant co-occurrence links with *Deinococcus, Hymenobacter, Methylobacterium, Rhodococcus*, and *Sphingomonas*, identifying it as the hub genus of the sexually mature microbiome. Notably, *Variovorax* abundance was positively correlated with expression of the male sex-determining gene amh (*r* = 0.42, *P* < .001^***^), and two of its network partners—*Sphingomonas* (*r* = 0.45, *P* < .001^***^) and *Deinococcus* (*r* = 0.23, *P* = .029^*^)—also showed positive correlations with amh. Comparable correlations were observed at the species level (Supplementary Fig. S12). For the female-associated gene *vtgr*, a positive correlation was observed with *Cetobacterium* (*r* = 0.238, *P* = .013^*^) (Supplementary Table S2). Together, these results indicate that *Variovorax* is the central hub of a co-occurring bacterial community that is correlated with male sex determination in eels.

### Culture-dependent identification of sex steroid-metabolizing gut microbes

LEfSe and network analysis revealed several bacterial genera associated with mature eels, potentially linked to host hormone production. To validate their sex steroid-metabolizing function (transformation or catabolism), we cultivated eel gut microbiota using culture-dependent approaches. We tested microbial steroid metabolism under strictly anaerobic, denitrifying, and microaerobic conditions. No sex steroids-metabolizing anaerobes were identified from the eel gut microbiota. By contrast, we collected 66 bacterial strains, all of which were capable of metabolizing sex steroids under microaerobic conditions (Table 1; Supplementary Dataset S4).

**Table 1:** A summary of hormone-metabolizing gut microbes (55 strains isolated from eel gut microbiota and 11 commercially purchased *Deinococcus* strains) identified in this present study. Individual bacterial strains were identified by 16S rRNA gene sequencing and BLAST analysis against NCBI reference genomes. Steroid metabolism was evaluated using phosphate-buffered chemically defined medium containing 1 mM testosterone, 11-ketotestosterone, or estradiol as the sole carbon source. Strain-level data are provided in Supplementary Dataset S4.

Number of bacterial isolates	Bacterial genera	Bacterial class	Major testosterone metabolites	Major 11-ketotestosterone metabolites	Major estradiol metabolites
7	*Gordonia*	Actinomycetes	AD, ADD, NP, UC	NP, UC	E1, NP
1	*Microbacterium*	Actinomycetes	AD	UC	E1
2	*Mycobacterium*	Actinomycetes	NP	NP	E1
5	*Mycolicibacterium*	Actinomycetes	NP	NP, UC	E1, NP
4	*Nocardia*	Actinomycetes	NP	NP, UC	E1, NP
2	*Rhodococcus*	Actinomycetes	NP	NP	E1
1	*Cytobacillus*	Bacilli	AD	NA	E1
1	*Paenibacillus*	Bacilli	AD	UC	E1
11	*Deinococcus*	Deinococci	AD, UC	NP	E1
2	*Brucella*	Alphaproteobacteria	AD	NP	E1, UC
1	*Devosia*	Alphaproteobacteria	NP	NA	NP
1	*Neotabrizicola*	Alphaproteobacteria	AD	NA	NA
2	*Paracoccus*	Alphaproteobacteria	AD	UC	E1
2	*Qipengyuania*	Alphaproteobacteria	NP	NP	NP
5	*Sphingomonas*	Alphaproteobacteria	NP	NP	E1, NP
1	*Stappia*	Alphaproteobacteria	NA	NP	E1
1	*Ectopseudomonas*	Gammaproteobacteria	NA	NA	NP
1	*Metapseudomonas*	Gammaproteobacteria	NP	UC	E1
13	*Pseudomonas*	Gammaproteobacteria	AD, ADD, DT, NP	UC	E1, NP, UC
1	*Pseudoxanthomonas*	Gammaproteobacteria	AD	UC	NP
1	*Shewanella*	Gammaproteobacteria	AD	UC	E1
1	*Stutzerimonas*	Gammaproteobacteria	NA	NA	E1, UC

Abbreviations: AD, 4-androstene-3,17-dione; ADD, androsta-1,4-diene-3,17-dione; DT, 1-dehydrotestosterone; E1, estrone; NA, no activity; NP, no product detected; UC, uncharacterized product.

These sex steroids-metabolizing bacterial isolates belonged to 22 bacterial genera: *Brucella, Cytobacillus, Deinococcus, Devosia, Ectopseudomonas, Gordonia, Metapseudomonas, Microbacterium, Mycobacterium, Mycolicibacterium, Neotabrizicola, Nocardia, Paenibacillus, Paracoccus, Pseudomonas, Pseudoxanthomonas, Qipengyuania, Rhodococcus, Shewanella, Sphingomonas, Stappia*, and *Stutzerimonas.* To elucidate their steroid metabolic pathways, we cultivated the bacterial isolates with three major sex steroids (testosterone, 11-ketotestosterone, and estradiol) under microaerobic conditions. After 3-day cultivation, we extracted microbial products from bacterial cultures using ethyl acetate and analyzed the extracts through UPLC-HRMS.

Steroid profile analysis revealed that most bacterial isolates performed oxidation reactions on the C-1/C-2 and/or 17-hydroxyl groups of the three sex steroids, maintaining the intact four-ring structure. The major microbial products included 4-androsten-3,17-dione (AD), androsta-1,4-diene-3,17-dione (ADD), 1-dihydrotestosterone (DT), and estrone (E1). A subset of gut microbes partially degraded testosterone, 11-ketotestosterone, and/or estradiol via uncharacterized pathways, yielding unknown products (UC; full strain list in Supplementary Dataset S4). In contrast, other isolates fully degraded the tested steroids with no products detectable in ethyl acetate extracts (NP; Supplementary Dataset S4, marked in red). Notably, numerous bacterial species from the taxa Actinomycetes and Alphaproteobacteria demonstrated the capacity to completely degrade 11-ketotestosterone, the primary androgenic hormone in teleost fish. In contrast, most bacterial isolates belonging to Gammaproteobacteria can metabolize testosterone but lack the enzymatic capability to degrade 11-ketotestosterone or estradiol.

### Functional genomics reveals the prevalence of steroid catabolic genes among eel gut bacterial isolates

**Figure 6:**
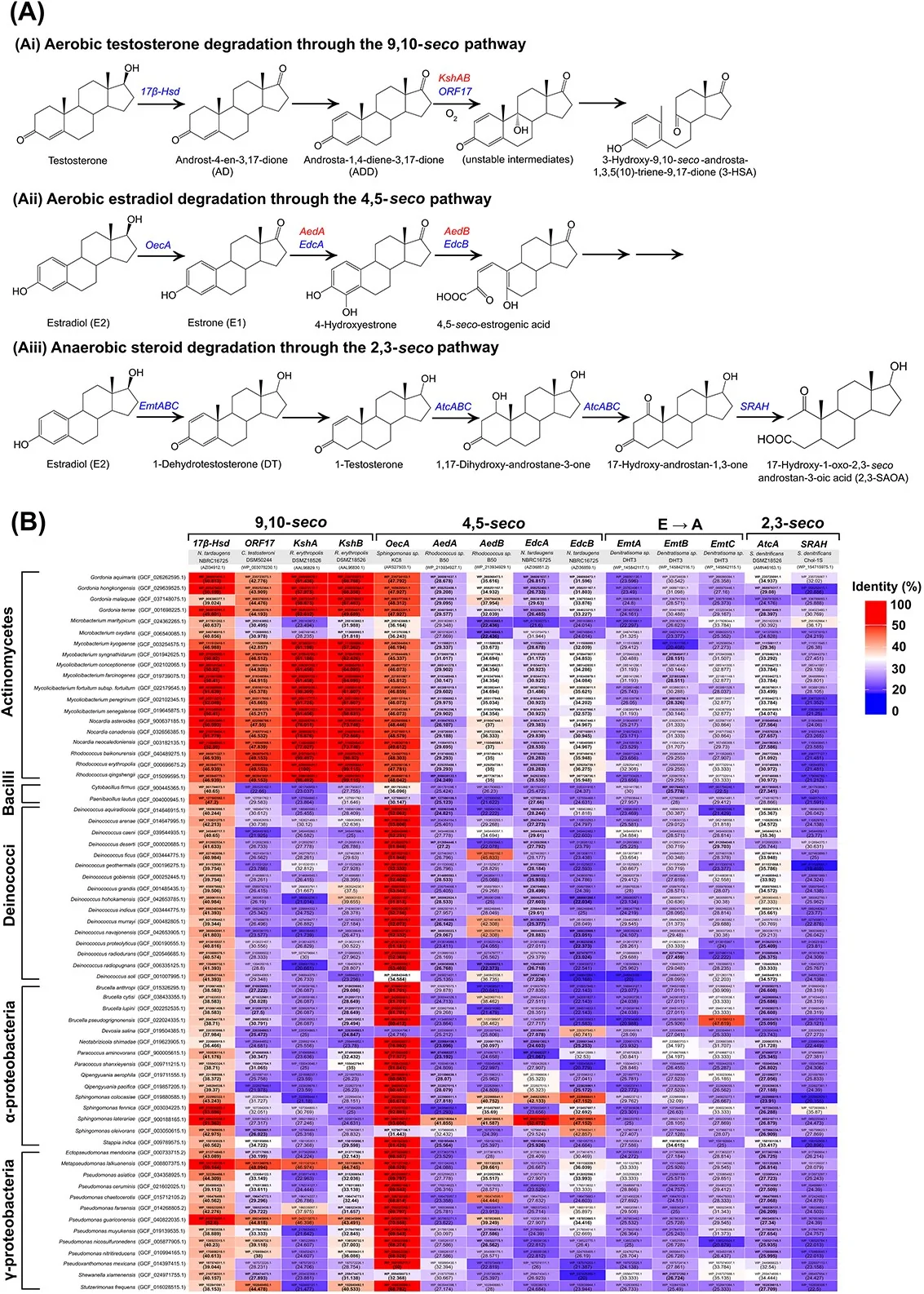
Distribution of steroid hormone metabolism genes in bacterial isolates from *Anguilla bicolor*. (A) Established degradation pathways for testosterone and estradiol in bacteria. (Ai) Aerobic testosterone degradation via the 9,10-*seco* pathway; (Aii) aerobic estradiol degradation via the 4,5-*seco* pathway; (Aiii) anaerobic steroid degradation via the 2,3-*seco* pathway. Red and blue annotations indicate enzymes from actinobacteria and proteobacteria, respectively. (B) Gene prevalence heatmap across selected bacterial genomes. Steroid metabolism genes were identified using BLAST searches with sequences from biochemically characterized enzymes or putative genes identified through comparative genomics. Reference sequences (deduced amino acid sequences): 9,10-*seco* pathway genes include 17*β*-*hsd* (AZI34912.1) from *Novosphingobium tardaugens* NBRC16725, ORF17 (WP_003078230.1) from *Comamonas testosteroni* DSM50244, and *kshA/kshB* (AAL96829.1/AAL96830.1) from *Rhodococcus erythropolis*; 4,5-*seco* pathway genes include *oecA* (ARS27503.1) from *Sphingomonas* sp. KC8, *aedA/aedB* (WP_213934927.1/WP_213934929.1) from *Rhodococcus* sp. B50, and *edcA/edcB* (AZI36851.2/AZI36859.1) from *N. tardaugens* NBRC16725; anaerobic estrogen-to-androgen biotransformation genes include *emtA/B/C* (WP_145842117.1/WP_145842116.1/WP_145842115.1) from *Denitratisoma* sp. DHT3; and 2,3-seco pathway genes include *atcA* (AMN46163.1) from *Steroidobacter denitrificans* DSMZ18526 and *SRAH* (WP_067170571.1) from *S. denitrificans* Chol-1S. The color gradient (blue-white-red) represents sequence identity percentage. Bold text indicates protein sequences with ≥ 90% coverage.

Steroid degradation capabilities in eel gut microbiota are primarily limited to Actinomycetes, Alphaproteobacteria, and Gammaproteobacteria. These bacterial taxa employ oxygen-dependent pathways for steroid degradation that have been characterized at the molecular level. Despite low sequence homology (deduced amino acid sequence identities < 40%), these bacteria utilize the oxygen-dependent 4,5-seco and 9,10-seco pathways to degrade estradiol and testosterone, respectively (Fig. 6A).

We assessed the distribution of characterized steroid metabolism genes across genomes from isolated eel gut microbes and closely related species (16S rRNA sequence identity ≥ 98.7%) (Fig. 6B). The *17β-hsd* gene, encoding 17*β*-hydroxysteroid dehydrogenase, shows widespread distribution across these genomes. Actinobacteria and Proteobacteria utilize this enzyme to convert testosterone to androstenedione (AD) [34, 35]. We identified homologous genes in *Deinococcus* species, whose gene products demonstrate the highest sequence similarity to proteobacterial enzymes (up to 42.2%) and actinomycetes enzymes (up to 52.6%). In contrast, genes involved in androgenic core-ring degradation (including *ORF17, kshA*, and *kshB*) show limited distribution, primarily confined to Proteobacteria and Actinomycetes. Among Deinococci, the *ksh* genes were identified only in select species such as *Deinococcus grandis* and *D. hohokamensis*.

For estradiol degradation, both Actinobacteria [36, 37] and Alphaproteobacteria [38, 39] employ the 4,5-*seco* pathway. The *oecA* gene, involved in converting estradiol to estrone, shows broad distribution across bacterial genomes, including Deinococci (with deduced amino acid sequence identity up to 53.8%). However, genes encoding estrogenic A-ring degradation enzymes (*aedA, aedB, edcA*, and *edcB*) are restricted to Alphaproteobacterial and Actinobacterial genomes (Fig. 6B), suggesting that Bacilli and Deinococci species lack aerobic estrogen degradation capabilities.

Anaerobic degradation of sex steroids has been documented in only a few Beta- and Gamma-proteobacteria [35, 40]. Using characterized degradation genes from *Denitratisoma* sp. strain DHT3 as queries, we detected limited distribution of these genes among eel gut bacteria. Our comparative genomic analysis thus indicates that anaerobic steroid metabolism is not prevalent in the eel gut microbiome.

## Discussion

In the present study, we specifically investigated gut microbiota changes during sexual differentiation rather than sexual maturation (silvering) for three key reasons. First, silvering involves dramatic gut regression and atrophy as eels prepare for nonfeeding oceanic migration, fundamentally altering the intestinal environment [19]. Second, the natural fasting behavior during this phase causes significant reductions in gut microbial diversity and density due to the absence of dietary substrates and deteriorating intestinal habitat. Third, the gut microbiota's contribution to host physiology may diminish during silvering, when eels cease feeding and rely entirely on stored energy rather than active digestion and metabolism. Even so, other microbial functions—such as the utilization of host-derived substrates (e.g. mucus, sloughed epithelial cells, and lipid derivatives)—may persist and warrant further investigation. By examining actively feeding, sexually differentiating eels, we captured biologically relevant host–microbiota interactions at a critical life stage where these relationships are most functionally significant and experimentally informative.

### Sex steroid profiles in shortfin eels

Our study provides important insights into the sex steroid profiles of shortfin eels (*A. bicolor*) cultivated under controlled freshwater conditions. The results demonstrate that serum testosterone levels in male shortfin eels were comparable to those in mature females, while serum estradiol levels in females were significantly higher than in males. However, we observed notably limited production of 11-ketotestosterone in both mature male and female eels under these freshwater conditions. The low 11-ketotestosterone levels observed in our study likely reflect the artificial freshwater cultivation environment rather than a fundamental species difference from Japanese eels. 11-Ketotestosterone is well-established as the primary androgen in teleost fish, including multiple eel species [41, 42], and its production is typically enhanced during sexual maturation and environmental transitions [43, 44]. In natural conditions, eels undergo complex hormonal changes during their catadromous migration from freshwater to marine environments for reproduction [20, 45]. The artificially maintained freshwater conditions in our study may have suppressed the normal elevation of 11-ketotestosterone that would occur during natural sexual maturation and seawater adaptation.

Environmental factors, particularly salinity, strongly influence steroid hormone production in eels [43, 46, 47]. Because the transition to seawater typically stimulates gonadotropin release and 11-ketotestosterone production [47], our freshwater-maintained eels likely reflect an incomplete maturation state rather than the full reproductive hormone profile of naturally maturing individuals.

### Enterohepatic circulation of sex steroids in eels

Sex steroid levels and GSI vary significantly across vertebrate species. Our findings demonstrate that *A. bicolor* females exhibit substantially elevated GSI and estradiol levels compared to other vertebrates. Specifically, these eels show GSI values of 2.5%–3.0% and E2 concentrations of 1.1–2.1 ng/ml, which are markedly higher than those observed in healthy female rats (GSI: 0.038%, E2: 0.012 ng/ml) [48, 49] and female mice (GSI: ~ 0.15%, E2: ~ 0.01 ng/ml) [50, 51].

The anatomical differences between eels and mammals may contribute to these elevated hormone levels. While mammals possess complex digestive systems with distinct small and large intestine divisions, eels have relatively simple digestive tracts—essentially a tube extending from the esophagus to the anus. The gut microbial communities of *A. bicolor* further distinguish these eels from mammals. While human gut microbiota are dominated by anaerobic bacteria (primarily Bacteroidota and Bacillota), eel gut communities are predominantly composed of aerobic or microaerophilic organisms, reflecting the dominance of Pseudomonadota and Actinomycetota. Although some obligate and facultative anaerobes (Bacteroidota and Bacillota) are present in eels, they represent a smaller fraction of the total community. This aerobic bias is further supported by our inability to isolate any anaerobic sex hormone-metabolizing bacteria from eel gut samples.

The eel intestine harbors a greater abundance of aerobic hormone-metabolizing bacteria, including *Deinococcus, Rhodococcus*, and *Sphingomonas*, compared to human and mouse gut microbial communities [23, 24, 52]. These specialized microbial communities actively metabolize the sex hormones produced by the eel’s enlarged gonads (testosterone, 11-ketotestosterone, and estradiol), generating various hormone metabolites such as androstenedione, androstenediol, estrone, and uncharacterized hormone metabolites. The unique anatomical features of the eel digestive system may facilitate enterohepatic circulation of these steroid compounds. The relatively simple gut architecture potentially allows bacterial hormone metabolites to cross the intestinal barrier more readily and enter systemic circulation. This hypothesis is supported by previous research demonstrating that steroids can traverse the fish intestinal barrier and participate in enterohepatic circulation [53, 54].

The elevated hormone levels, combined with the presence of specialized metabolizing bacteria and permeable gut architecture, suggest that eels might point to an efficient enterohepatic recycling system for sex steroids. Future investigations should focus on characterizing the biological activity of these bacterial hormone metabolites and elucidating their specific physiological roles in eel reproduction and development. Understanding this enterohepatic circulation pathway could provide valuable insights into the unique reproductive strategies of anguillid eels and their remarkable life cycle adaptations.

### Developmental changes in eel gut microbiota composition

Our microbiomics analyses reveal significant shifts in gut microbiota composition throughout eel development, with distinct patterns emerging across life stages and between sexes. Early developmental stages, including glass eels and elvers, are dominated by the bacterial phylum Pseudomonadota. In contrast, sex-determined male and female eels exhibit increased abundance of Deinococcota, indicating a major compositional transition during sexual development.

Notable sex-specific differences emerge in sex-determined eels. Female eels harbor higher proportions of facultative anaerobes, primarily from the phylum Bacillota, while males maintain elevated levels of aerobic and microaerophilic bacteria, predominantly Pseudomonadota. Key bacterial genera in sex-determined eels include *Variovorax, Deinococcus, Sphingomonas*, and *Rhodococcus*. Particularly intriguing is the positive correlation between the anti-Müllerian hormone gene (*amh*), a male-specific marker, and the abundance of *Sphingomonas, Variovorax*, and *Deinococcus*. This correlation suggests potential functional interactions between these bacterial taxa and eel sexual development pathways.

The observed microbiota shifts likely reflect changes in gut physiology during eel development. As eels grow, their intestinal length increases substantially [55, 56], potentially altering the gut microenvironment from subnormoxic to hypoxic conditions. This environmental transition subsequently influences both microbiota composition and metabolic functions. Our data demonstrate that glass eels, elvers, sex-undetermined eels, and male adults maintain guts dominated by aerobic and microaerophilic microbes. In contrast, female guts shift toward facultative anaerobe dominance, possibly reflecting the longer gut length and altered oxygen availability in reproductively active females. These findings align with previous research showing that Bacillota abundance positively correlates with fish body size, while Pseudomonadota abundance decreases in larger fish [57]. The progressive changes in gut oxygen availability during eel development appear to be key drivers of both microbial community structure and functional capacity, ultimately shaping the unique hormone-metabolizing capabilities observed in different life stages and sexes.

### Sex steroid metabolism by eel gut microbiota

Our study provides the first comprehensive characterization of sex steroid-metabolizing gut bacteria in teleost fish. We successfully isolated and identified 66 bacterial strains capable of metabolizing sex hormones, thereby validating the functional predictions derived from our microbiomics analyses. Multiple species from the genera *Rhodococcus* and *Sphingomonas* demonstrated robust aerobic steroid metabolism capabilities, which aligns with the high prevalence of steroid degradation genes detected in their genomes.

The eel gut microbiome’s steroid metabolism differs fundamentally from mammalian systems. Mammalian colons represent oxygen-depleted, highly reduced environments (approximately −200 mV) dominated by strictly anaerobic bacteria such as *Clostridium* species, which utilize sex steroids as electron acceptors [24]. In contrast, the short intestinal tracts of eels create a microaerobic ecosystem that supports the activity of oxygenases and corresponding aerobic microorganisms. This unique environment enables complete aerobic degradation of estradiol, a process that is predominantly reported under oxygen-rich conditions.

Isolating bacterial strains capable of complete estradiol degradation as single cultures remains a significant challenge in environmental microbiology, particularly from complex matrices such as sewage, wastewater, and estuarine sediments. In sewage and wastewater systems, bacterial communities are typically dominated by Betaproteobacteria, followed by Gammaproteobacteria and Actinomycetes [58]. Estuarine environments show a different pattern, with Gammaproteobacteria predominating, followed by Alphaproteobacteria and Actinomycetes [59]. Most bacteria isolated from these environments achieve only partial estradiol degradation, typically converting it to intermediate metabolites like estrone, which may persist and retain estrogenic activity [60, 61]. For instance, activated sludge isolates frequently convert estradiol to estrone but fail to mineralize estrone further, with only rare strains demonstrating complete degradation and growth on estradiol as the sole carbon source [61]. Furthermore, degradation efficiencies are often enhanced in bacterial consortia rather than single isolates, indicating that complete estradiol mineralization typically requires cooperative processes involving multiple microbial species with complementary metabolic capabilities [60–62].

The isolation of multiple bacterial genera from the eel gut capable of completely degrading estradiol as single isolates represents a significant finding that provides novel insights into microbial estradiol catabolism. The bacterial genera we isolated—*Gordonia, Mycolicibacterium, Nocardia* (Actinomycetes), *Devosia, Qipengyuania, Sphingomonas* (Alphaproteobacteria), and *Ectopseudomonas, Pseudomonas, Pseudoxanthomonas* (Gammaproteobacteria)—are consistent with taxa previously implicated in steroid degradation. However, these genera are rarely reported as single isolates capable of fully mineralizing estradiol [60, 61, 63]. The ability of these eel gut bacteria to utilize estradiol as a sole carbon and energy source suggests specialized metabolic adaptations. These adaptations may be linked to the unique ecological niche of the eel gut, where steroid hormones are likely present at elevated concentrations due to the host's distinctive physiology and reproductive biology. This finding highlights the potential of fish gut microbiomes as sources of novel steroid-degrading bacteria with biotechnological applications.

### The gut microbe *Deinococcus* spp. as newly identified steroid metabolizers

A particularly significant finding in our study involves the physiological and molecular characterization of *Deinococcus* species from the eel gut, which showed increasing abundance from glass eel to adult stages. While *Deinococcus* spp. have been previously found in European eel guts and characterized as microaerophiles growing optimally in hypoxic environments [29, 64], our study provides the first evidence of their capability for aerobic steroid metabolism. *Deinococcus* belongs to phylum Deinococcota, comprising three genera: *Deinococcus, Deinobacterium*, and *Truepera* [65]. As radiation-resistant extremophiles, these species have been isolated from diverse environments, including wastewater systems [66]. Despite their ecological significance, steroid hormone metabolic pathways in *Deinococcus* remain poorly understood, with only *D. actinosclerus* previously identified as capable of partial estradiol oxidation (with estrone as the major product) via *oecA*-encoded 3*β*,17*β*-hydroxysteroid dehydrogenase [67]. Consistently, most *Deinococcus* species in our study simply oxidized the 17-hydroxy group of sex steroids, causing mild loss in hormonal activity. However, at least one species, namely *Deinococcus ficus*, exhibited remarkable differential metabolic capabilities toward structurally related androgens. While testosterone was only partially metabolized, producing androstenedione and uncharacterized steroidal metabolites, 11-ketotestosterone underwent complete degradation. This substrate specificity suggests distinct metabolic pathways for these androgens. This differential capability highlights the substrate-specific nature of bacterial steroid metabolism and suggests that 11-ketotestosterone may represent a preferred carbon source for this isolate, providing the first evidence of host–microbe interactions between eel sex hormones and steroid-metabolizing *Deinococcus* while advancing our understanding of sex hormone metabolism in eel-associated gut microbiota.

### Implications for host–microbe interactions and eel development

Our findings suggest that interactions between the eel gut microbiota and their hosts are bidirectional. The aerobic bacteria we identified (e.g. *Sphingomonas* and *Rhodococcus*) likely consume gut sex steroids, potentially decreasing circulating levels of androgens and estrogens in their hosts. This may negatively impact eel development and sexual maturation. As these bacterial species often inhabit aquatic environments and represent dominant microorganisms in aquaculture, removing sex steroid-degrading bacteria from the eel gut and surrounding environments may facilitate the development and sexual maturation of captive eels.

Recent studies using mouse models have demonstrated that testosterone-degrading bacteria can significantly decrease serum testosterone levels through enterohepatic circulation [23]. Similarly, these accumulated bacteria might continually influence cultivated eels in aquaculture ponds, creating a feedback loop that affects hormonal balance and reproductive development. Conversely, wild eels might be less influenced by the same bacteria due to multiple environmental factors, such as salinity, temperature, and environmental hormones. For example, environmental hormones coexist in habitats of benthic eels. Higher concentrations of hormones may alter microbiota compositions and the population density of various hormone-degrading bacteria. Both environmental hormones and bacteria may influence benthic fishes’ growth and maturation in downstream rivers. This may explain why large variations in gut microbiota appear in aquatic animals, such as wild benthic eels.

## Conclusion

This study demonstrates that host–gut microbiota interactions play crucial roles in developmental programming and sexual differentiation of *A. bicolor*. The identification of novel steroid-metabolizing bacteria, particularly *Deinococcus* species with unique substrate specificities, expands our understanding of microbial steroid metabolism and host–microbe interactions in aquatic vertebrates. Through our microbiomics approach, we predicted the steroid hormone metabolizing function of several bacterial genera through correlation analyses and validated their functions through culture-dependent approaches. Microbial co-occurrence network analysis shows that the abundance of *Deinococcus, Sphingomonas*, and *Variovorax* was positively correlated with each other and may interact for synergistic hormone metabolism. This metabolic specificity suggests the potential application of these bacterial genera as biomarkers for eel sexual development.

The isolation of multiple bacterial genera capable of completely degrading estradiol as single isolates has important biotechnological implications. These bacteria could be utilized for bioremediation of endocrine-disrupting compounds in aquatic environments or for developing novel bioprocesses for steroid hormone degradation. Additionally, understanding the specific mechanisms of steroid metabolism in these bacteria could inform strategies for managing hormone levels in aquaculture settings.

## Supplementary Material

Supplementary_material_ycag241

## Data Availability

The PacBio raw data generated during the current study are available in the NCBI Sequence Read Archive (SRA) repository at https://www.ncbi.nlm.nih.gov/sra?linkname=bioproject_sra_all&from_uid=1244584, and can be assessed with accession numbers ranging from SRR32945574 to SRR32945632. Processed data are provided as Supplementary Datasets: Supplementary Dataset S1—COI and 16S rRNA gene sequences; Supplementary Dataset S2—Species and genus count table; Supplementary Dataset S3—Nodes and edges of the microbial co-occurrence network; Supplementary Dataset S4—Steroid hormone-metabolizing activity of cultured bacterial isolates. Cultured isolates obtained in the culture-dependent experiments (Supplementary Dataset S4) are available from the corresponding author upon reasonable request.
