# Supplementary material for: Gut microbiomics reveals host–microbe crosstalk during sexual differentiation in the shortfin eel Anguilla bicolor
Source: ISME Commun. 2026 Aug 22;6(1):ycag241. doi: 10.1093/ismeco/ycag241 (PMC13584205; doi:10.1093/ismeco/ycag241)
Supplement: Supplementary file 1 [file supplementary_material_ycag241.zip › D-25-00394_Supplementary Materials_R3.pdf]

Supplementary Information for

Gut microbiomics reveals host-microbe crosstalk during sexual differentiation in the shortfin eel *Anguilla bicolor*

Guo-Jie Brandon-Mong, Tzong-Huei Lee, Yi-Lung Chen, Tsun-Hsien Hsiao, Guan-Lun Wu, Yi-Li Lai, Chieh-Yin Weng, Ronnie G Gicana, Yu-Ting Yen, Yi-Fan Xu, Tzi-Yuan Wang, and Yin-Ru Chiang

This PDF file includes the following:

SI Materials and Methods
SI References
Figs. S1 to S12
Tables S1 to S2

SI Materials and Methods

Eel culture system

Glass eels (*A. bicolor pacifica*, a subspecies of *A. bicolor*) were collected from an estuary in Hualien, eastern Taiwan, and transported to a fish farm with non-polluted stream water in Hsinchu, Taiwan. Day 0 was designated as the arrival date of glass eels at the farm. The cultivation process proceeded through three stages: 1) Glass eels (GL) were reared for 6 months in freshwater tanks (10-15 cm depth, 20-25°C); 2) Resulting elvers (EV, fingerlings) were maintained in 1-2 m depth tanks for 1 year; 3) sex-undetermined eels (SU) were grown in 2-3 m depth tanks for 2 years. Specimens were collected at four developmental stages: glass eels (GL; day 0), elvers (EV; 6 months), sex-undetermined eels (SU; 1.5 years), and sex-determined eels (females and males; 3 years).

Gut samples were collected from glass eels before first feeding. The remaining eels were fed bloodworms (*Chironomus circumdatus*) at the glass eel stage and commercial pellets from the elver stage onward. To minimise the acute dietary influence on gut microbiota composition, fish were starved for one week before sample collection. The proximate composition of the bloodworms (*Chironomus* sp.) was: crude protein ~50.6%, carbohydrate ~21.8%, lipid ~2.8%, crude fibre ~2.8%, and moisture ~85.8% [1]. The proximate composition of the commercial pellets was: crude protein >44%, carbohydrate <16.5%, lipid >9%, crude fibre <2%, and moisture <11% (https://www.tunglifeed.com/).

Detection and quantification of cholesterol and hormones

Bloodworm and feed pellet samples (5 g each) were extracted with ethyl acetate. After complete solvent evaporation, the residues were reconstituted in 1 mL of methanol. 10-fold diluted samples were analyzed by ultra-performance liquid chromatography–atmospheric pressure chemical ionization–high-resolution mass spectrometry (UPLC–APCI–HRMS). Contents of cholesterol, androgens (testosterone, dihydrotestosterone, androstenedione, androstadienedione), and estrogens (estrone, estradiol, estriol) were detected by comparing their retention times and m/z values with those of authentic standards. Cholesterol was quantified using the o-phthalaldehyde method [2].

Ethics statement

All procedures (Protocol ID: 22-11-1925, 22-11-1926) were approved by the Institutional Animal Care & Use Committee of Academia Sinica, Taiwan.

Sample collection

Forty-four A. bicolor eels at different developmental stages were sampled: glass eels (GL, n = 6), elvers (EV, n = 8), sex-undetermined eels (SU, n = 10), sex-determined males (M, n = 10), and sex-determined females (F, n = 10). Eels were euthanized via hypothermia using ice-slurry immersion, followed by morphological measurements (body weight and length). Blood samples were collected from the tail of sex-determined eels (M and F) in heparinized tubes, centrifuged (3000 rcf, 10 minutes), and the plasma was stored at -20°C for hormone quantification. Tissue samples were processed using tissue-specific protocols. Gonad tissue was preserved in RNAlater overnight at 4°C, then stored at -80°C until RNA extraction. Proximal intestine samples were

dissected to expose the luminal surface and immediately stored at -80°C prior to DNA extraction. Distal intestine samples were maintained on ice for same-day bacterial culture.

Eel hormone quantification

Plasma testosterone was quantified using the Testosterone Parameter Assay Kit (R&D Systems, Inc., USA). Undiluted plasma samples were analyzed following the manufacturer's protocol. Absorbance was measured at 450 nm with 570 nm wavelength correction. Concentrations were calculated using a 4-parameter logistic (4PL) regression model with standards ranging from 0 to 10 ng/mL. Plasma estradiol was measured using the Estradiol Parameter Assay Kit (R&D Systems, Inc., USA). Samples were diluted 1.6-fold according to protocol specifications. Absorbance was measured at 450 nm with 570 nm wavelength correction. Final concentrations were calculated using a 4PL regression model (standard range: 0 to 3 ng/mL) and adjusted for dilution factor. Plasma 11-KT concentrations were quantified using the 11-Ketotestosterone Competitive ELISA kit (Invitrogen, USA) with modifications. Briefly, 500 µl ethyl acetate (EA) was added to each 100 µl plasma sample in a 1.5 mL tube and vortexed for 10 minutes. Samples were centrifuged for 5 seconds, kept on ice, and the upper layer extracted. This extraction was repeated once to improve efficiency. Upper layer extracts from each sample were combined in a single 1.5 mL tube. EA was vacuum-dried and reconstituted in 110 µl assay buffer. Reagents and samples were added to wells, and absorbance was measured at 450 nm. 11-KT concentrations were calculated using a standard curve (0 to 2 ng/mL).

RT-qPCR analysis

Expression levels of anti-müllerian hormone (*amh*) and vitellogenin receptor (*vtgr*) genes were quantified using RT-qPCR. Total RNA was extracted from gonad tissue using Direct-zol RNA Kits (R2052, ZymoResearch) and purified with Turbo DNA-free Kit (Thermo Fisher Scientific). First-strand cDNA synthesis was performed using SuperScript® IV System with oligo-dT primers (Thermo Fisher Scientific). Gene expression was analyzed using Power SYBR® Green PCR Master Mix with qPCR primers [Forward (*vtgr*): 5'-GCTCATAGACCGCAAGACC-3'; Reverse (*vtgr*): 5'-GCCTTACACACGCCAGAAGT-3' [3]; Forward (*amh*): 5'-TCCTGGTCAGCACTGCGTATC-3'; Reverse (*amh*): 5'-TCCCGCACCGACAGACA-3'; Forward (ef1a): 5'-TGTGGGAGTCAACAAGATGGA-3' [4]; Reverse (ef1a): 5'-CTCAAAACGCTTCTGGCTGTA-3' [4] in a QuantStudioTM 5 Real-Time PCR System (Thermo Fisher Scientific), with elongation factor 1α (ef1a) serving as the internal control.

DNA extraction and 16S rDNA amplicon sequencing

Genomic DNA was extracted from five sample types (eel gut, freshwater, seawater, red worms, and fish feed pellets) using PowerFecal DNA isolation kit (Qiagen, Germany). The full-length 16S genes (V1-V9 regions) was amplified by barcoded 16S gene-specific primers. According to the Amplification of Full-Length 16S Gene with Barcoded Primers for Multiplexed SMRTbell Library Preparation and Sequencing Procedure (PacBio), each primer is designed to contain a 5' buffer sequence (GCATC) with a 5' phosphate modification, a 16-base barcode and the degenerate 16S gene-specific forward or reverse primer sequences. (Forward:5'Phos/GCATC- 16-base barcode - AGRGTTYGATYMTGGCTCAG -3', Reverse: 5'Phos/GCATC- 16-base barcode – RGYTACCTTGTTACGACTT -3'). Degenerate base identities are: R = A, G; Y = C, T; M = A, C. Degenerate primers were designed based on [5]. PCR amplification was performed in duplicate using SuperRed PCR Master Mix (BIOTOOLS Co., Ltd., Taiwan) with the following

touchdown protocol: Initial denaturation at 95 °C for 30 s; 16 cycles of 10 s at 95 °C, 30 s at 62 °C with 1 °C decreasing delta temperature every subsequent cycle (final temperature was 46 °C), 1 min at 72 °C; followed by 25 cycles of 10 s at 95 °C, 30 s at 46 °C, 1 min at 72 °C; and a final elongation step for 5 min at 72 °C. Successful amplification (~1,500 bp) was confirmed by 1.0% agarose gel electrophoresis. Purified PCR products (AMPure PB Beads) were sequenced using PacBio technology (BIOTOOLS Co., Ltd.). 16S rRNA gene sequence data are available in the Sequence Read Archive (SRA) under BioProject ID PRJNA1244584, with accession numbers ranging from SRR32945574-SRR32945632.

Eel species identification

To identify eel species, the cytochrome oxidase subunit 1 (*COI*) gene was PCR amplified from DNA samples using SuperRed PCR Master Mix and universal primers (Forward LCO1490: 5'-GGTCAACAAATCATAAAGATATTGG-3' and Reverse HCO2198: 5'-TAAACTTCAGGGTGACCAAAAAATCA-3') described by Folmer et al. (1994) [6]. The PCR followed a touchdown protocol: Initial denaturation at 95 °C for 30 s; 16 cycles of 10 s at 95 °C, 30 s at 62 °C with 1 °C decreasing delta temperature every subsequent cycle (final temperature was 46 °C), 1 min at 72 °C; followed by 40 cycles of 10 s at 95 °C, 30 s at 46 °C, 1 min at 72 °C; and a final elongation step for 5 min at 72 °C. Successful amplification (~700 base pairs) was confirmed using 1.0% agarose gel electrophoresis. After the purification of the PCR products, Sanger sequencing was performed, and a maximum likelihood phylogenetic tree was constructed using MEGA software [7]. The analysis included NCBI *COI* reference sequences from seven eel species (*A. japonica* [LC782003.1, KU168670.1], *A. marmorata* [MZ435985.1, MW283183.1], *A. anguilla* [KU927487.1, KU927488.1], *A. bicolor pacifica* [LC548771.1, MT647227.1], A. *bicolor bicolor* [PP352530.1, MZ312365.1], and *A. rostrata* [MW822312.1, MW822295.1]), with *Conger* (MN711446.1) serving as an outgroup for comparison. For bloodworm samples, The analysis included NCBI *COI* reference sequences from 6 worm species (*Chironomus circumdatus* [KY845435.1, OP927187.1], *Chironomus dilutes* [MF708827.1, PP418460.1], *Chironomus plumosus* [OP927630.1, PQ179804.1], *Chironomus riparius* [MK584516.2, MT535087.1], *Chironomus yoshimatsui* [LC773146.1, LC773370.1], with *Polypedilum nubifer* (MN521311.1) serving as an outgroup for comparison.

Gut microbiota analysis

Raw PacBio reads were processed using QIIME2 pipeline [8]. Sequences were filtered to remove: 1) Those outside 1000-1600 bp length range; 2) Primers and chimeric sequences; 3) sequences with ambiguous bases; 4) Non-bacterial sequences (chloroplast, eukaryote, mitochondria, unknown taxa). Sequence quality was assessed using QIIME2 View (stats.qzv). Filtered sequences were denoised using DADA2 to generate amplicon sequence variants (ASVs) [9]. Species-level taxonomic classification used bacterial 16S rRNA RefSeq sequences from NCBI nucleotide database, with taxonomic data retrieved via NCBI Datasets Command Line Tool. Taxonomic classification thresholds were: Species: ≥98.7%; Genus: ≥94.5%; Family: ≥86.5%; Order: ≥82.0%; Class: ≥78.5%; Phylum: ≥75.0% [10-12]. ASVs were binned into taxonomic count tables using Mothur phylotype command [13]. Rarefaction curve analysis was conducted to validate the adequacy of sequencing depth for gut microbiota analysis. Samples were rarefied to 2,499 reads for diversity analyses, including Shannon's and Simpson’s diversity index for alpha diversity and Bray-Curtis dissimilarity for beta diversity. The LEfSe-based analysis (lefser) was conducted to identify biomarkers (e.g., gut microbial genera) that statistically differentiate sex-determined eels

(M & F) and younger eels (GL, EV & SU) [14, 15]. Within lefser, a non-parametric Kruskal-Wallis test was conducted to detect gut bacterial genera with significant differential abundance between the two groups [14]. Finally, Linear Discriminant Analysis (LDA) was performed in lefser to estimate the effect size of each differentially abundant genus and identify biomarkers with large differences between groups [14]. To identify bacterial taxa shared across all developmental stages, a Venn diagram analysis was performed at both the genus and species levels using presence–absence data, with a taxon considered present when its mean abundance exceeded zero. Core taxa were defined as those with a prevalence above 80%.

Correlation analysis between gene expression and gut microbiota

Two correlation analyses were performed: 1) Among gut bacterial taxa (genus and species levels) across developmental stages; 2) Between sex-specific gene expression (*amh* & *vtgr*) and gut bacterial taxa (genus and species levels) across eel life stages (GL, EV, SU, M, F). Bacterial taxa with a mean relative abundance below 0.2% were removed to minimize spurious correlations. Pairwise correlations between bacterial genera were computed using FastSpar with 20 iterations. Statistical significance was assessed by generating 10,000 bootstrap replicates, each processed with 5 iterations, and p-values were calculated using fastspar_pvalues. Correlations with $p < 0.05$ were retained for network construction [16, 17]. Significant correlations ($p < 0.05$) were used to calculate network centrality scores (degree, closeness, betweenness, and eigenvector) using Gephi [18]. For the network interpretations, each node represents a microbial genus, with node color and size indicating the eigenvector centrality score (red: higher score, blue: lower score). The edge color represents the correlation strength between nodes, reflecting correlations between microbial taxa. Correlations between microbial taxa and the expression of *amh* and *vtgr* are indicated as correlation strengths (r values). Correlation coefficients (r) were interpreted as weak (0.1-0.3), moderate (0.3-0.7), or strong (0.7-1.0). Asterisks indicate significance levels for correlations between microbial genera and the expression of *amh* and *vtgr*: $^{*}p < 0.05$; $^{**}p < 0.01$; $^{***}p < 0.001$.

Culture-dependent approaches for isolation of hormone-metabolizing bacteria

(i) Microbial isolation strategy

There were three sequential strategies for isolating steroid-metabolizing microbes. First, intestinal microbiota was cultivated under three physiological conditions: aerobic, anaerobic, and fermentative growth, with testosterone, 11-ketotestosterone or estradiol provided as the specific steroids in all conditions. Second, single bacterial colonies were isolated from Tryptic Soy Agar (TSA) plates to obtain pure cultures. Finally, the steroid-metabolizing activities of each isolate were validated using silica-gel 60 F254 thin-layer chromatography (TLC) (Merck). The details are as follows:

(ii) Gut microbiota cultivation

Eel intestines were dissected, placed in sterile Petri dishes, and longitudinally opened under aseptic conditions. The tissue was suspended in sterile saline (1:10 w/v), vortexed to release microbes, and allowed to settle for 1 min to remove debris. The supernatant was pooled for inoculation. Intestinal microbiota were inoculated under three conditions: (1) aerobic culture: 1 mL suspension was inoculated into the phosphate-buffered chemically defined medium [19, 20]; (2) denitrifying culture: 1 mL suspension was inoculated into a denitrification medium [21]; (3) anaerobic fermentation: 4 mL suspension was added to an anaerobic bottle, flushed with nitrogen for 30 min, and inoculated into DCB-1 medium [22]. All cultures were supplemented with 1 mM of

testosterone, 11-ketotestosterone or estradiol and incubated at 30°C, 100 rpm, with sub-samples collected on days 0, 1, 3, 5, 7, 14 and 21.

(iii) Determination of microbial steroid metabolic capability through TLC assay

Steroid of each inoculation was extracted via ethyl acetate and the concentrations were monitored using TLC plates. Once depletion was observed, serial dilutions (10^1–10^5) were spread on TSA for isolation. Single colonies with distinct morphologies were subcultured and purified through quadrant-streaking method. Each isolate was inoculated in phosphate-buffered chemically defined medium [19, 20] supplemented with 1 mM of testosterone, 11-ketotestosterone or estradiol at 30°C with shaking at 100 rpm. Sub-sampling was performed on days 0, 3, and 7, with culture extracts obtained using ethyl acetate. Steroid conversion by each isolate was verified by spotting the ethyl-acetate extracts onto TLC plates and comparing the disappearance of parent steroids and appearance of reference standards under UV illumination.

(iv) Functional validation with reference *Deinococcus* strains

In this present study, we could not isolate any *Deinococcus* species from the eel gut microbiota. To confirm the steroid metabolism capability of *Deinococcus* species, 11 *Deinococcus* strains were purchased from the Bioresource Collection and Research Center (BCRC), Hsinchu, Taiwan, including *Deinococcus radiodurans* (BCRC num: 12827T; ATCC13939), *Deinococcus radiopugnans* (12856T; ATCC19172), *Deinococcus grandis* (17376T; ATCC43672), *Deinococcus proteolyticus* (17377T; MRP), *Deinococcus geothermalis* (17378T; DSM11300), *Deinococcus* indicus (17379T; DR1), *Deinococcus murrayi* (17380T; DSM11303), *Deinococcus deserti* (17541T; VCD115), *Deinococcus navajonensis* (17558T; CGMCC1.12543), *Deinococcus hohokamensis* (17559T; CGMCC4.7563), and *Deinococcus ficus* (17568T; CC-FR2-10). Link: https://catalog.bcrc.firdi.org.tw/KeywordResult?searchType=1&key=deinococcus&bno=&strain all=all. Each strain was cultured in R2A medium and purified through quadrant streaking. Then, single colony was cultured in phosphate-buffered chemically defined medium [19, 20] with 1% oxygen with either testosterone, 11-ketotestosterone or estradiol for 7 days. Steroid degradation was monitored using TLC.

Steroid-metabolizing isolates were identified through 16S rRNA gene sequencing. Genomic DNA was extracted using the Presto Mini gDNA Bacteria Kit, followed by PCR amplification of the 16S rRNA gene using primers 27F (5'-AGAGTTTGATCMTGGCTCAG-3') and 1492R (5'-TACGGYTACCTTGTTACGACTT-3') [5]. The amplified products were sequenced using the Sanger method, and the resulting sequences were analyzed using BLASTn against the NCBI database for taxonomic identification.

Functional genomic analysis

In total, we isolated 55 gut bacterial strains with testosterone/estradiol metabolism activity from the eels. Among these, 47 strains were identified as known bacterial species (≥98.7% 16S rRNA gene identity; see Dataset S4 for details). We identified *Deinococcus* in the eel gut and purchased 11 *Deinococcus* species from BCRC. Bacterial genomes from Actinomycetes (n = 19), Bacilli (n = 2), Deinococci (n = 11), Alphaproteobacteria (n = 15), and Gammaproteobacteria (n = 13) were retrieved from the NCBI database for comparative functional genomic analysis. Genomes were analyzed by local BLAST against reference sequences; characterized enzyme protein sequences involve in testosterone or estradiol metabolism. Reference sequences included: 1) 9,10-seco pathway: 17β-hsd (AZI34912.1) of *Novosphingobium tardaugens* NBRC16725 [23], ORF17 (WP_003078230.1) of *Comamonas testosteroni* DSM50244 [24], kshA (AAL96829.1) and kshB (AAL96830.1) of *Rhodococcus erythropolis* [25]; 2) 4,5-seco pathway: oecA (ARS27503.1) of

Sphingomonas sp. KC8 [26], aedA (WP_213934927.1) and aedB (WP_213934929.1) of *Rhodococcus* sp. strain B50 [27, 28], and edcA (AZI36851.2) and edcB (AZI36859.1) of *Novosphingobium tardaugens* NBRC16725 [23]; 3) Estrogen → Androgen pathway: emtA (WP_145842117.1), emtB (WP_145842116.1) and emtC (WP_145842115.1) of *Denitratisoma* sp. strain DHT3 [29]; 4) 2,3-seco pathway: atcA (AMN46163.1) of *Steroidobacter denitrificans* DSMZ18526 [30] and SRAH (WP_067170571.1) of *Steroidobacter denitrificans* Chol-1S [31]. BLAST results were visualized in a heatmap showing protein sequence identity between bacterial genomes and reference sequences. Color gradient (blue-white-red) indicates sequence identity. Protein sequences with ≥90% coverage (followed Duan et al. 2024) [32] are shown in bold text.

Statistical Analysis

Data normality was assessed using the Shapiro-Wilk test. Data with normal distributions were analyzed using Welch's t-test; non-normal distributions using Wilcoxon rank sum test. Significance was set at $p < 0.05$. Bacterial analyses included: 1) Differential abundance: lefser [14] based on LEfSe [15]; 2) Alpha diversity: two-sample t-test; 3) Beta diversity: PERMANOVA for bacterial community structure.

SI References

1. Žák J, Roy K, Dyková I, Mráz J, Reichard M. Starter feed for carnivorous species as a practical replacement of bloodworms for a vertebrate model organism in ageing, the turquoise killifish *Nothobranchius furzeri*. J Fish Biol 2022;**100**:894-908. https://doi.org/10.1111/jfb.15021

2. Chiang YR, Ismail W, Müller M, Fuchs G. Initial steps in the anoxic metabolism of cholesterol by the denitrifying *Sterolibacterium denitrificans*. J Biol Chem 2007;**282**:13240-9. https://doi.org/10.1074/jbc.M610963200

3. Morini M, Lafont AG, Maugars G, Baloche S, Dufour S, Asturiano JF et al. Identification and stable expression of vitellogenin receptor through vitellogenesis in the European eel. *Animal* 2020;**14**:1213-22. https://doi.org/10.1017/s1751731119003355

4. Lin CJ, Jeng SR, Lei ZY, Yueh WS, Dufour S, Wu GC et al. Involvement of transforming growth factor beta family genes in gonadal differentiation in Japanese eel, *Anguilla japonica*, according to sex-related gene expressions. *Cells* 2021;**10**:3007. https://doi.org/10.3390/cells10113007

5. Lane DJ. 16S/23S rRNA sequencing. In: Stackebrandt E, Goodfellow M (eds.), Nucleic Acid Techniques in Bacterial Systematics. John Wiley & Sons, 1991, 115-75.

6. Folmer O, Black M, Hoeh W, Lutz R, Vrijenhoek R. DNA primers for amplification of mitochondrial cytochrome C oxidase subunit I from diverse metazoan invertebrates. *Mol Mar Biol Biotechnol* 1994;**3**:294-9.

7. Tamura K, Stecher G, Kumar S. MEGA11: Molecular Evolutionary Genetics Analysis Version 11. *Mol Biol Evol* 2021;**38**:3022-7. https://doi.org/10.1093/molbev/msab120

8. Hall M, Beiko RG. 16S rRNA gene analysis with QIIME2. *Methods Mol Biol* 2018;**1849**:113-29. https://doi.org/10.1007/978-1-4939-8728-3_8

9. Callahan BJ, McMurdie PJ, Holmes SP. Exact sequence variants should replace operational taxonomic units in marker-gene data analysis. *ISME J* 2017;**11**:2639-43. https://doi.org/10.1101/113597

10. Barco RA, Garrity GM, Scott JJ, Amend JP, Nealson KH, Emerson D. A genus definition for bacteria and archaea based on a standard genome relatedness index. *mBio* 2020;**11**:e02475-19. https://doi.org/10.1128/mbio.02475-19

11. Chun J, Oren A, Ventosa A, Christensen H, Arahal DR, da Costa MS et al. Proposed minimal standards for the use of genome data for the taxonomy of prokaryotes. *Int J Syst Evol Microbiol* 2018;**68**:461-6. https://doi.org/10.1099/ijsem.0.002516

12. Yarza P, Yilmaz P, Pruesse E, Glöckner FO, Ludwig W, Schleifer KH et al. Uniting the classification of cultured and uncultured bacteria and archaea using 16S rRNA gene sequences. *Nat Rev Microbiol* 2014;**12**:635-45. https://doi.org/10.1038/nrmicro3330

13. Schloss PD, Westcott SL, Ryabin T, Hall JR, Hartmann M, Hollister EB et al. Introducing mothur: open-source, platform-independent, community-supported software for describing and comparing microbial communities. *Appl Environ Microbiol* 2009;**75**:7537-41. https://doi.org/10.1128/aem.01541-09

14. Khleborodova A, Gamboa-Tuz SD, Ramos M, Segata N, Waldron L, Oh S. lefser: implementation of metagenomic biomarker discovery tool, LEfSe, in R. *Bioinformatics* 2024;**40**:btae707. https://doi.org/10.1093/bioinformatics/btae707

15. Segata N, Izard J, Waldron L, Gevers D, Miropolsky L, Garrett WS et al. Metagenomic biomarker discovery and explanation. *Genome Biol* 2011;**12**:R60. https://doi.org/10.1186/gb-2011-12-6-r60

16. Friedman J, Alm EJ. Inferring correlation networks from genomic survey data. *PLoS Comput Biol* 2012;**8**:e1002687. https://doi.org/10.1371/journal.pcbi.1002687

17. Watts SC, Ritchie SC, Inouye M, Holt KE. FastSpar: rapid and scalable correlation estimation for compositional data. *Bioinformatics* 2019;**35**:1064-6. https://doi.org/10.1093/bioinformatics/bty734

18. Bastian M, Heymann S, Jacomy M. Gephi: an open source software for exploring and manipulating networks. In: International AAAI Conference on Weblogs and Social Media. Association for the Advancement of Artificial Intelligence, 2009. https://doi.org/10.1609/icwsm.v3i1.13937

19. Chen YL, Yu CP, Lee TH, Goh KS, Chu KH, Wang PH et al. Biochemical mechanisms and catabolic enzymes involved in bacterial estrogen degradation pathways. *Cell Chem Biol* 2017;**24**:712-24. https://doi.org/10.1016/j.chembiol.2017.05.012

20. Hsiao TH, Lee TH, Chuang MR, Wang PH, Meng M, Horinouchi M et al. Identification of essential β-oxidation genes and corresponding metabolites for oestrogen degradation by actinobacteria. *Microb Biotechnol* 2022;**15**:949-66. https://doi.org/10.1111/1751-7915.13921

21. Wei ST, Wu YW, Lee TH, Huang YS, Yang CY, Chen YL et al. Microbial functional responses to cholesterol catabolism in denitrifying sludge. *mSystems* 2018;**3**:e00113-18. https://doi.org/10.1128/msystems.00113-18

22. Hemme CL, Fields MW, He Q, Deng Y, Lin L, Tu Q et al. Correlation of genomic and physiological traits of thermoanaerobacter species with biofuel yields. *Appl Environ Microbiol* 2011;**77**:7998-8008. https://doi.org/10.1128/aem.05677-11

23. Ibero J, Galán B, Rivero-Buceta V, García JL. Unraveling the 17β-estradiol degradation pathway in *Novosphingobium tardaugens* NBRC 16725. *Front Microbiol* 2020;**11**:588300. https://doi.org/10.3389/fmicb.2020.588300

24. Horinouchi M, Hayashi T, Yamamoto T, Kudo T. A new bacterial steroid degradation gene cluster in *Comamonas testosteroni* TA441 which consists of aromatic-compound degradation genes for seco-steroids and 3-ketosteroid dehydrogenase genes. *Appl Environ Microbiol* 2003;**69**:4421-30. https://doi.org/10.1128/aem.69.8.4421-4430.2003

25. van der Geize R, Hessels GI, van Gerwen R, van der Meijden P, Dijkhuizen L. Molecular and functional characterization of kshA and kshB, encoding two components of 3-ketosteroid 9alpha-hydroxylase, a class IA monooxygenase, in *Rhodococcus erythropolis* strain SQ1. *Mol Microbiol* 2002;**45**:1007-18. https://doi.org/10.1046/j.1365-2958.2002.03069.x

26. Zhang M, Gao S, Pan K, Liu H, Li Q, Bai X et al. Functional analysis, diversity, and distribution of the ean cluster responsible for 17β-estradiol degradation in sphingomonads. *Appl Environ Microbiol* 2024;**90**:e0197423. https://doi.org/10.1128/aem.01974-23

27. Tian K, Meng Q, Li S, Chang M, Meng F, Yu Y et al. Mechanism of 17β-estradiol degradation by *Rhodococcus equi* via the 4,5-seco pathway and its key genes. *Environ Pollut* 2022;**312**:120021. https://doi.org/10.1016/j.envpol.2022.120021

28. Hsiao TH, Chen YL, Meng M, Chuang MR, Horinouchi M, Hayashi T et al. Mechanistic and phylogenetic insights into actinobacteria-mediated oestrogen biodegradation in urban estuarine sediments. *Microb Biotechnol* 2021;**14**:1212-27. https://doi.org/10.1111/1751-7915.13798

29. Wang PH, Chen YL, Wei ST, Wu K, Lee TH, Wu TY et al. Retroconversion of estrogens into androgens by bacteria via a cobalamin-mediated methylation. *Proc Natl Acad Sci U S A* 2020;**117**:1395-403. https://doi.org/10.1073/pnas.1914380117

30. Yang FC, Chen YL, Tang SL, Yu CP, Wang PH, Ismail W et al. Integrated multi-omics analyses reveal the biochemical mechanisms and phylogenetic relevance of anaerobic androgen biodegradation in the environment. *ISME J* 2016;**10**:1967-83. https://doi.org/10.1038/ismej.2015.255

31. Jacoby C, Peller L, Wenzler J, Luttermann M, Seiche W, Breit B et al. Ring A cleaving beta-diketone hydrolase is a key enzyme of steroid degradation in anaerobic bacteria. *Environ Microbiol* 2025;**27**:e70034. https://doi.org/10.1111/1462-2920.70034

32. Duan Y, Santos-Júnior CD, Schmidt TS, Fullam A, de Almeida BLS, Zhu C et al. A catalog of small proteins from the global microbiome. *Nat Commun* 2024;**15**:7563. doi: 10.1038/s41467-024-51894-6

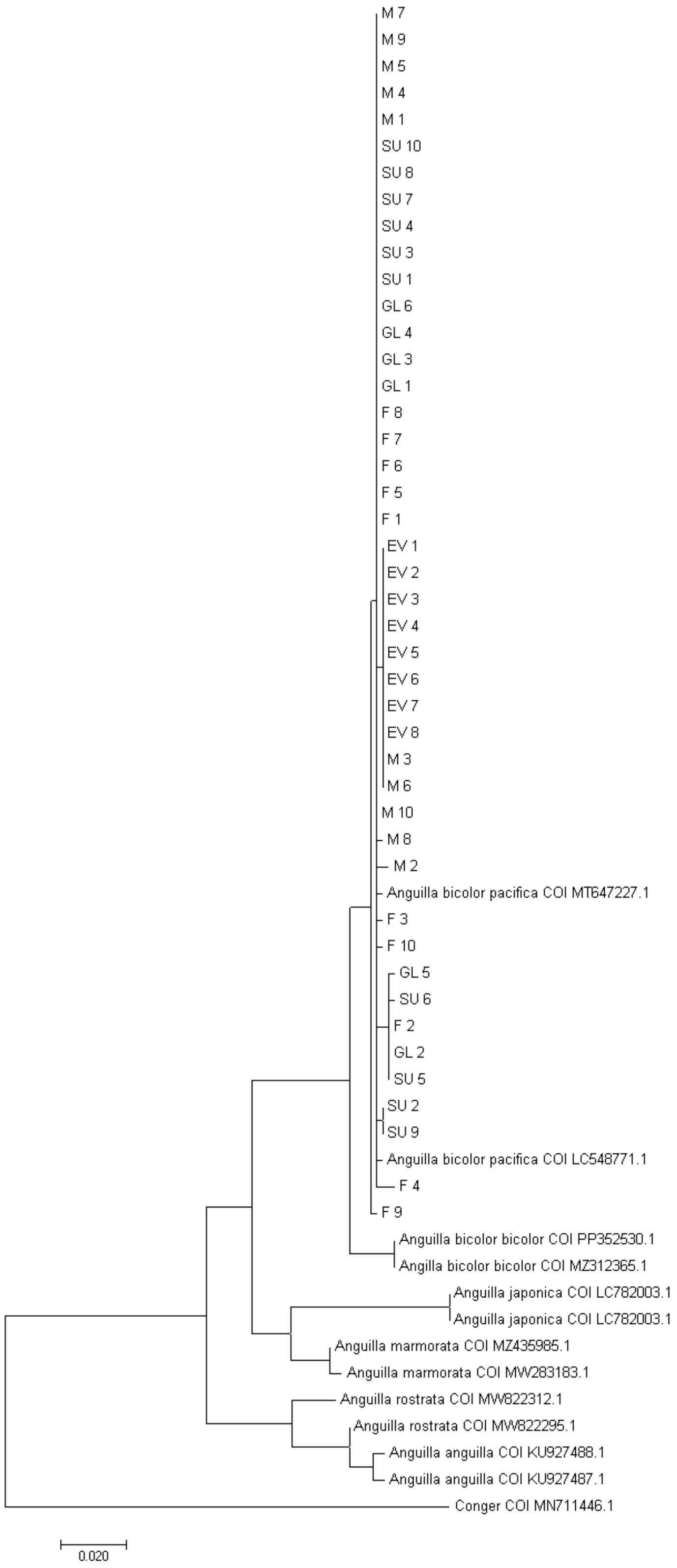

Supplemental Figure S1. Maximum likelihood phylogenetic tree based on *COI* sequences of *Anguilla bicolor pacifica* in this study and reference sequences. *Conger* served as an outgroup.

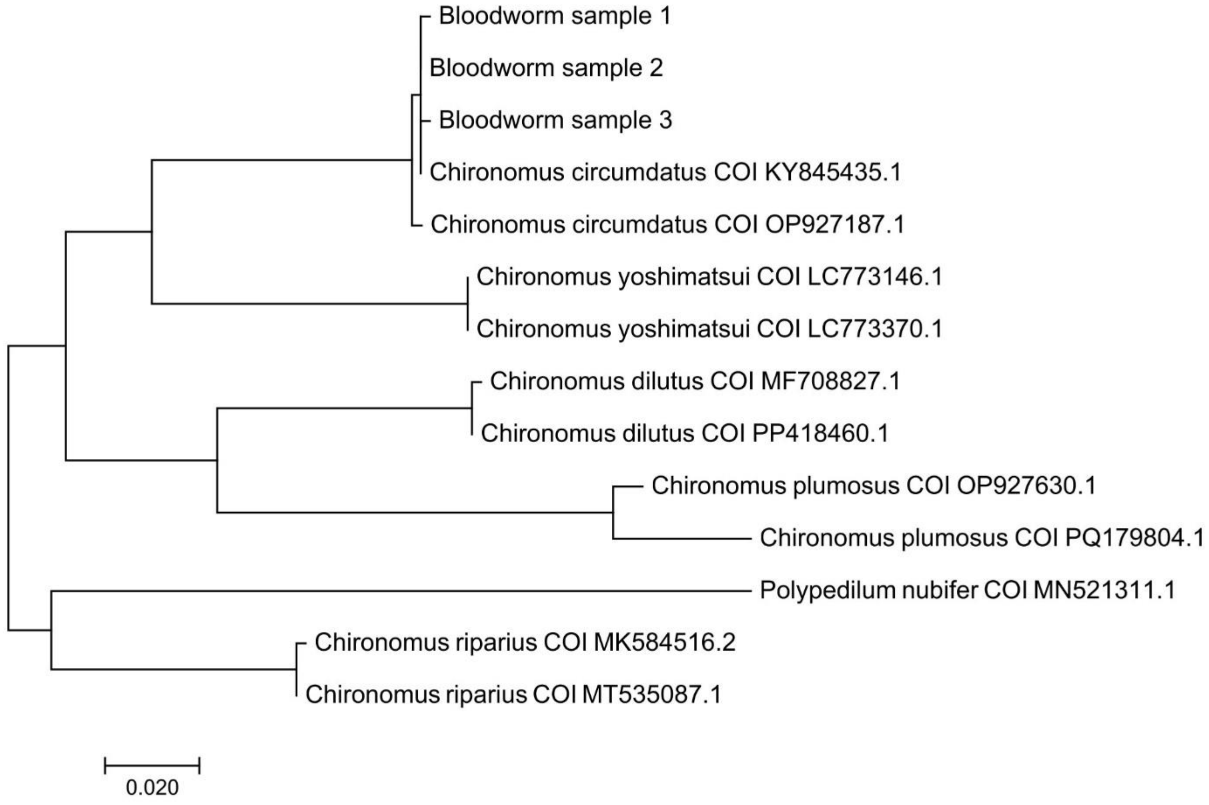

Supplemental Figure S2. Maximum likelihood phylogenetic tree based on *COI* sequences of bloodworm (*Chironomus* spp.) in this study and reference sequences. *Polypedilum nubifer* served as an outgroup.

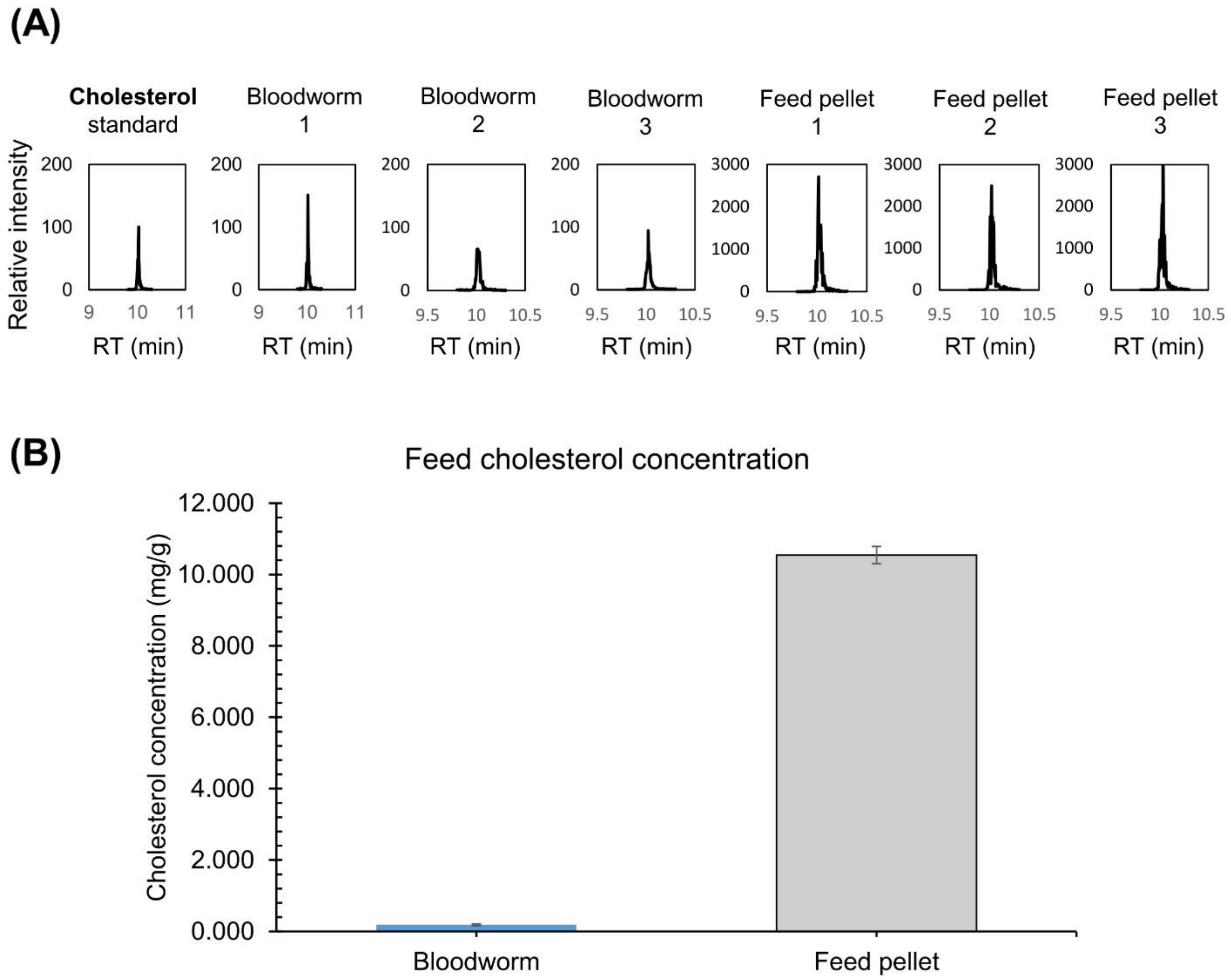

Supplemental Figure S3. Detection and quantification of cholesterol and hormones in feed. (A) Cholesterol was detected in both bloodworm and feed pellet samples, whereas androgens (testosterone, dihydrotestosterone, androstenedione, androstadienedione) and estrogens (estrone, estradiol, estriol) were below the detection limit by UPLC–APCI–HRMS. (B) Cholesterol content (mean ± SEM, n = 3) quantified in bloodworm and feed pellet samples using the o-phthalaldehyde method.

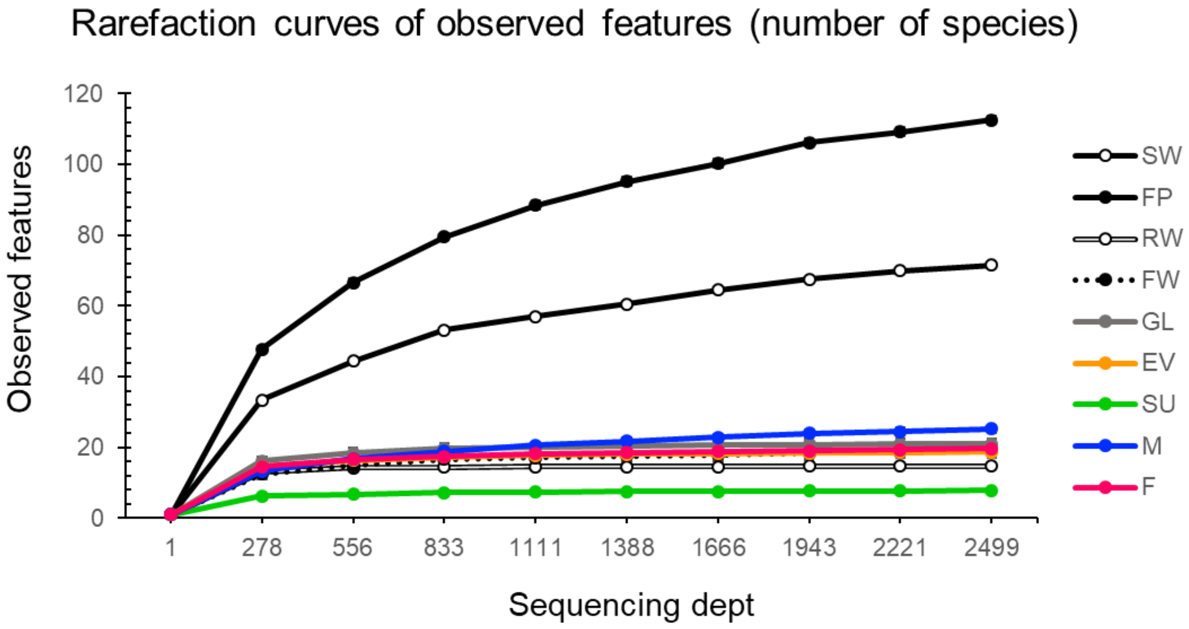

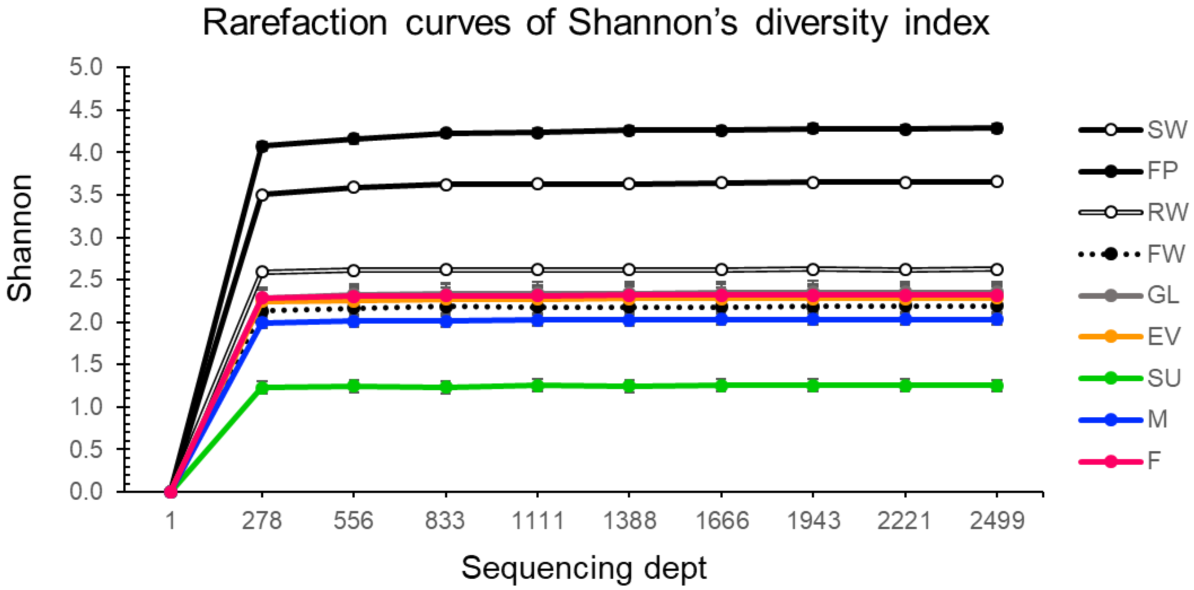

Supplemental Figure S4. Rarefaction curves of observed features (number of species) and Shannon's diversity index.

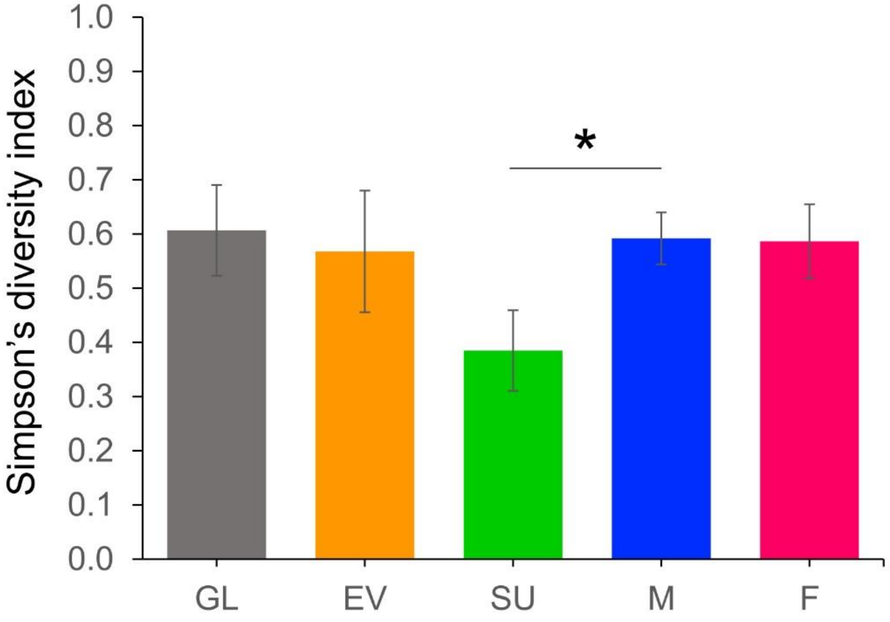

Supplemental Figure S5. Simpson's diversity index of the eel gut microbiota (genus level). Data were analyzed using Welch's t-test; *$p < 0.05$.

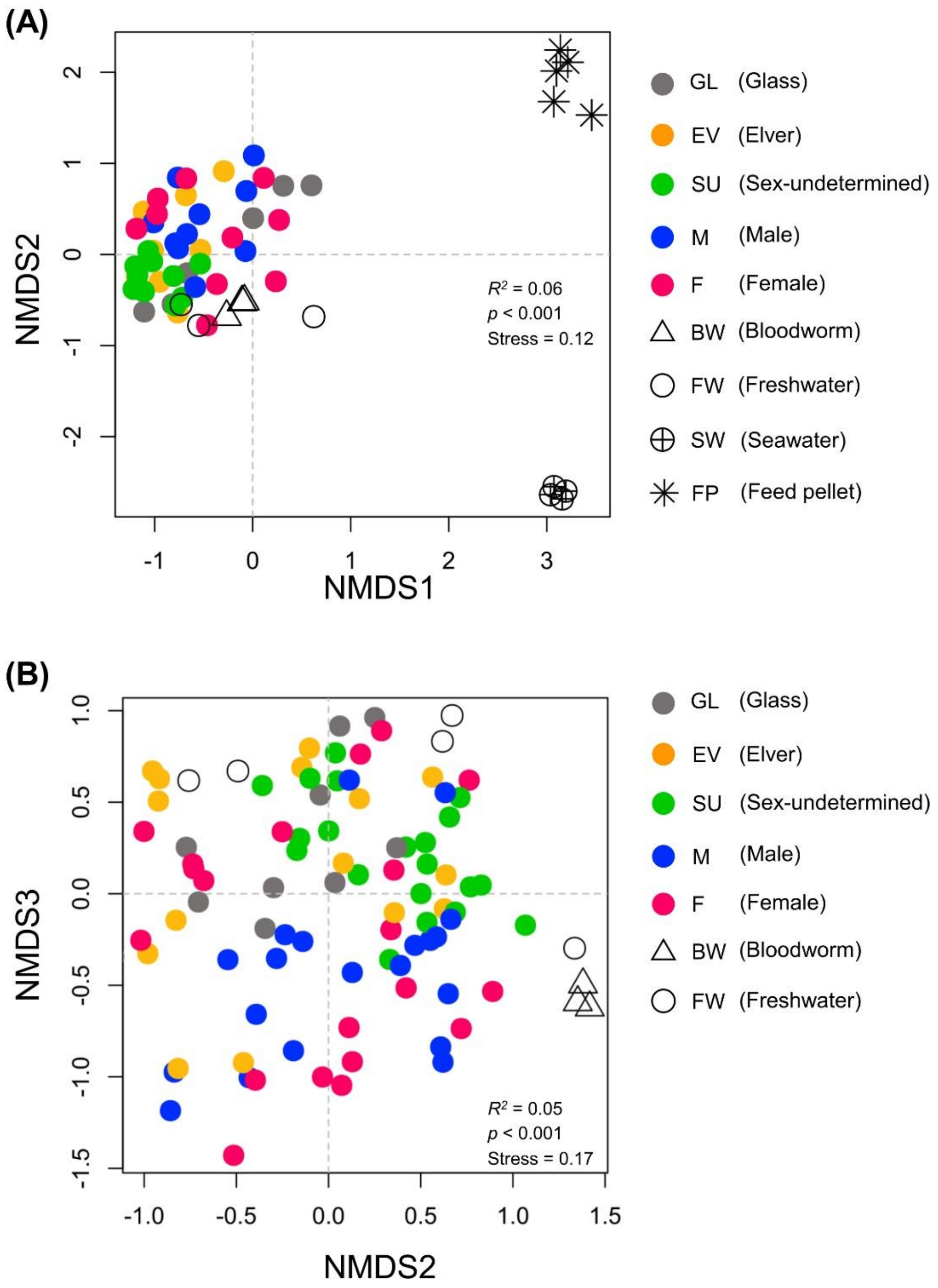

Supplemental Figure S6. Beta-diversity analysis of gut microbial communities and environmental controls at the genus level based on Bray-Curtis dissimilarity, visualized using NMDS. A) Gut microbial composition (GL, EV, SU, M, and F) and controls (RW: red worms; FW: freshwater; SW: seawater; FP: feed pellet). B) Gut microbial composition (GL, EV, SU, M, and F) with RW (red worms) and FW (freshwater).

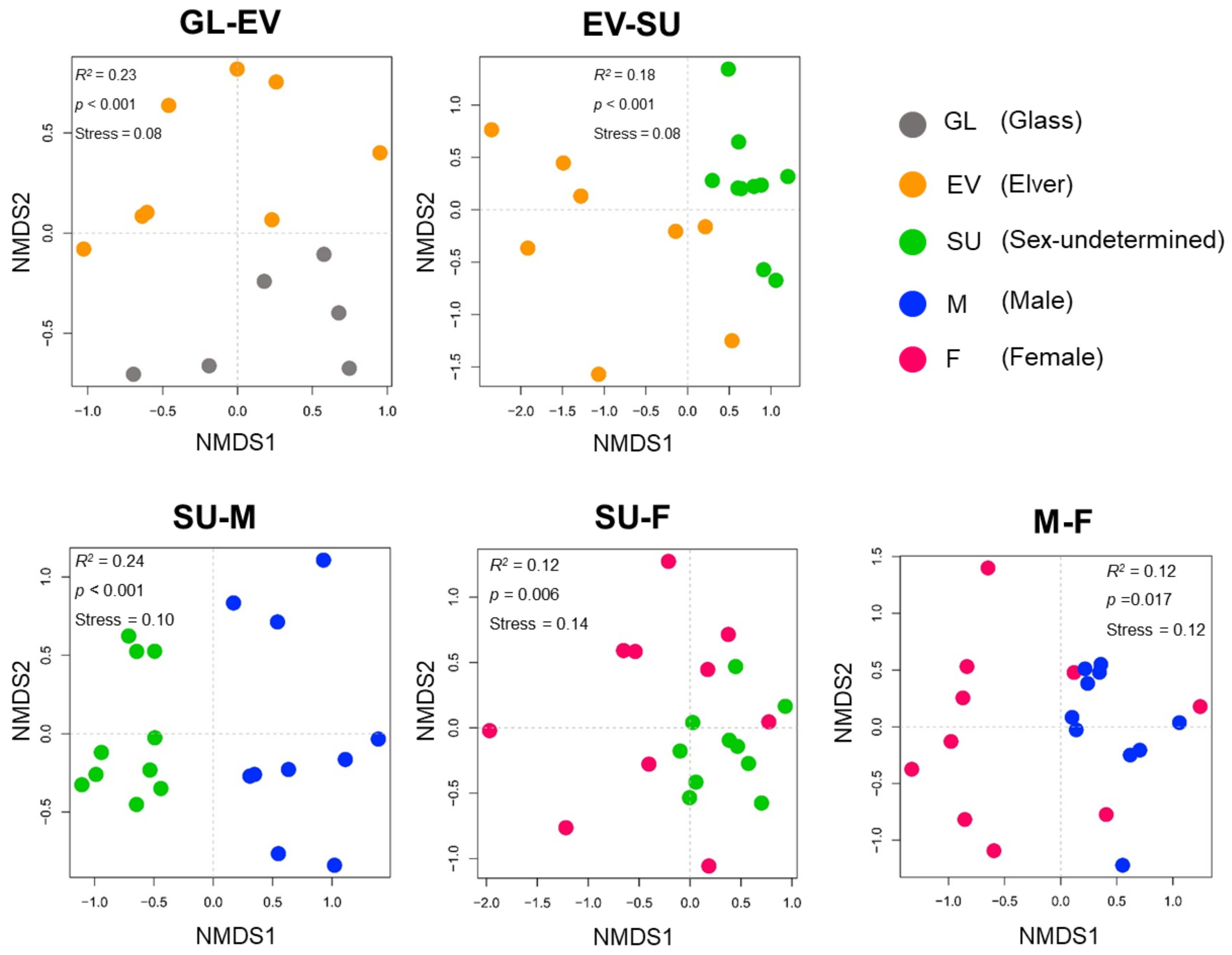

Supplemental Figure S7. Pairwise comparisons of gut microbial compositional structure between consecutive developmental stages (GL, EV, SU, M, and F) based on Bray-curtis dissimilarity and visualized in NMDS.

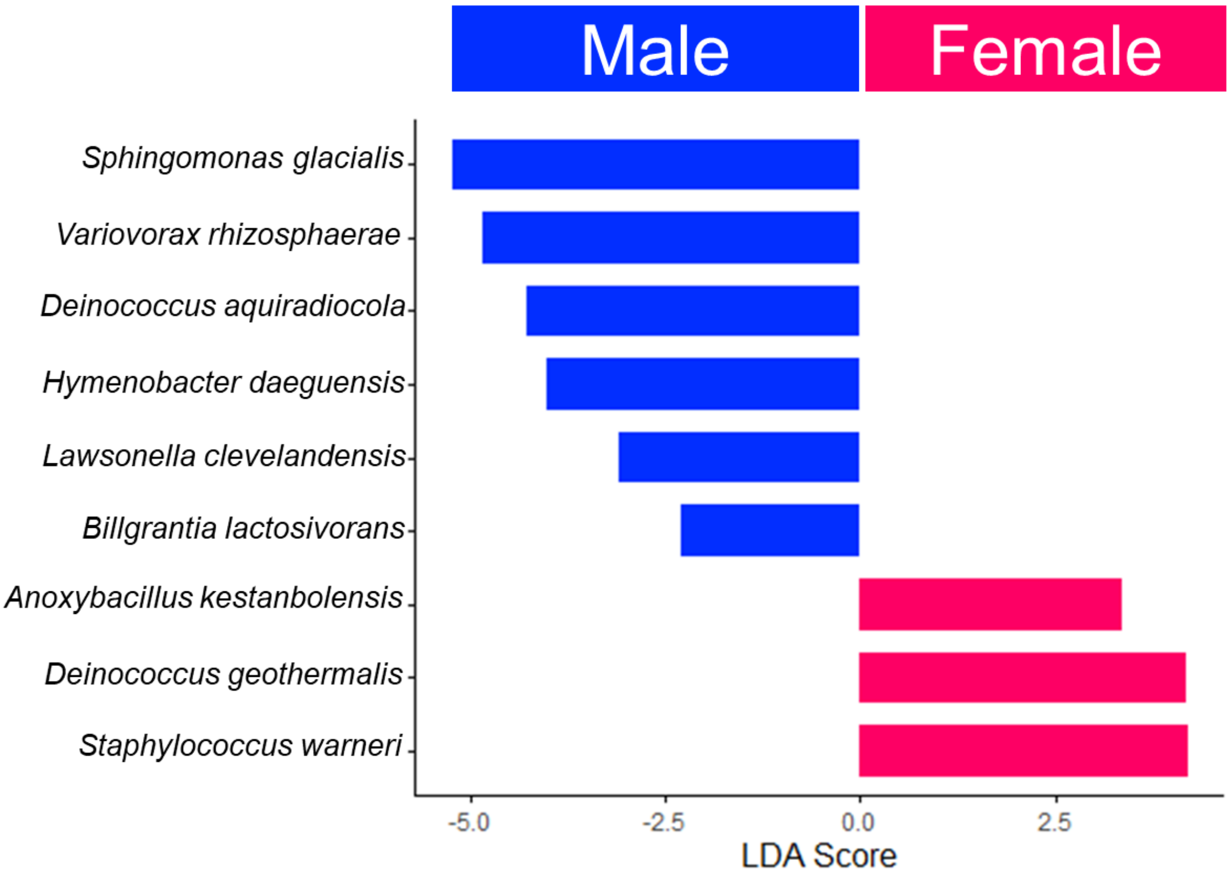

Supplemental Figure S8. LEfSe: Differential abundance of gut microbial genera between male and female eels.

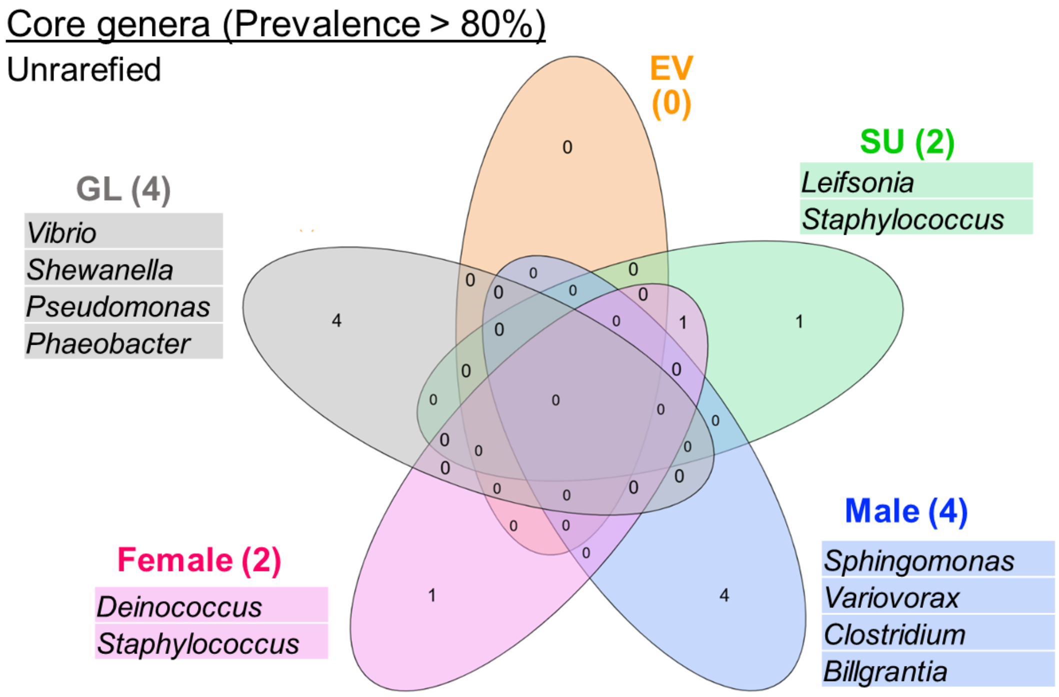

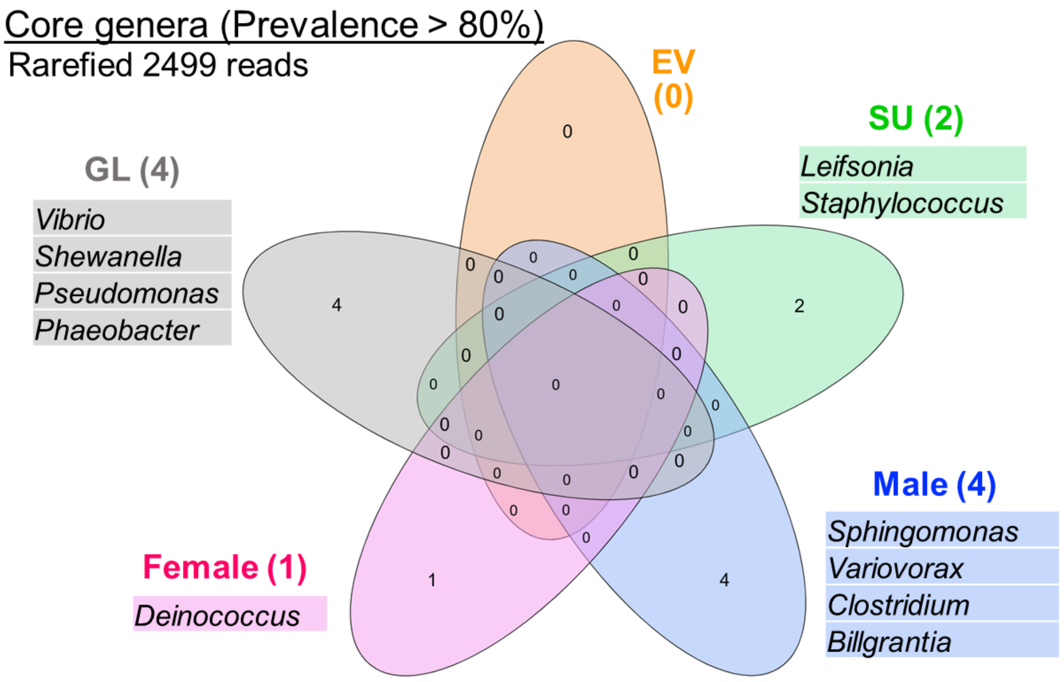

Supplemental Figure S9. Venn diagram analysis revealed no core genus (prevalence >80%) shared across all developmental stages in either unrarefied or rarefied count tables. Core genera identified within each developmental stage are listed in the corresponding Venn diagram.

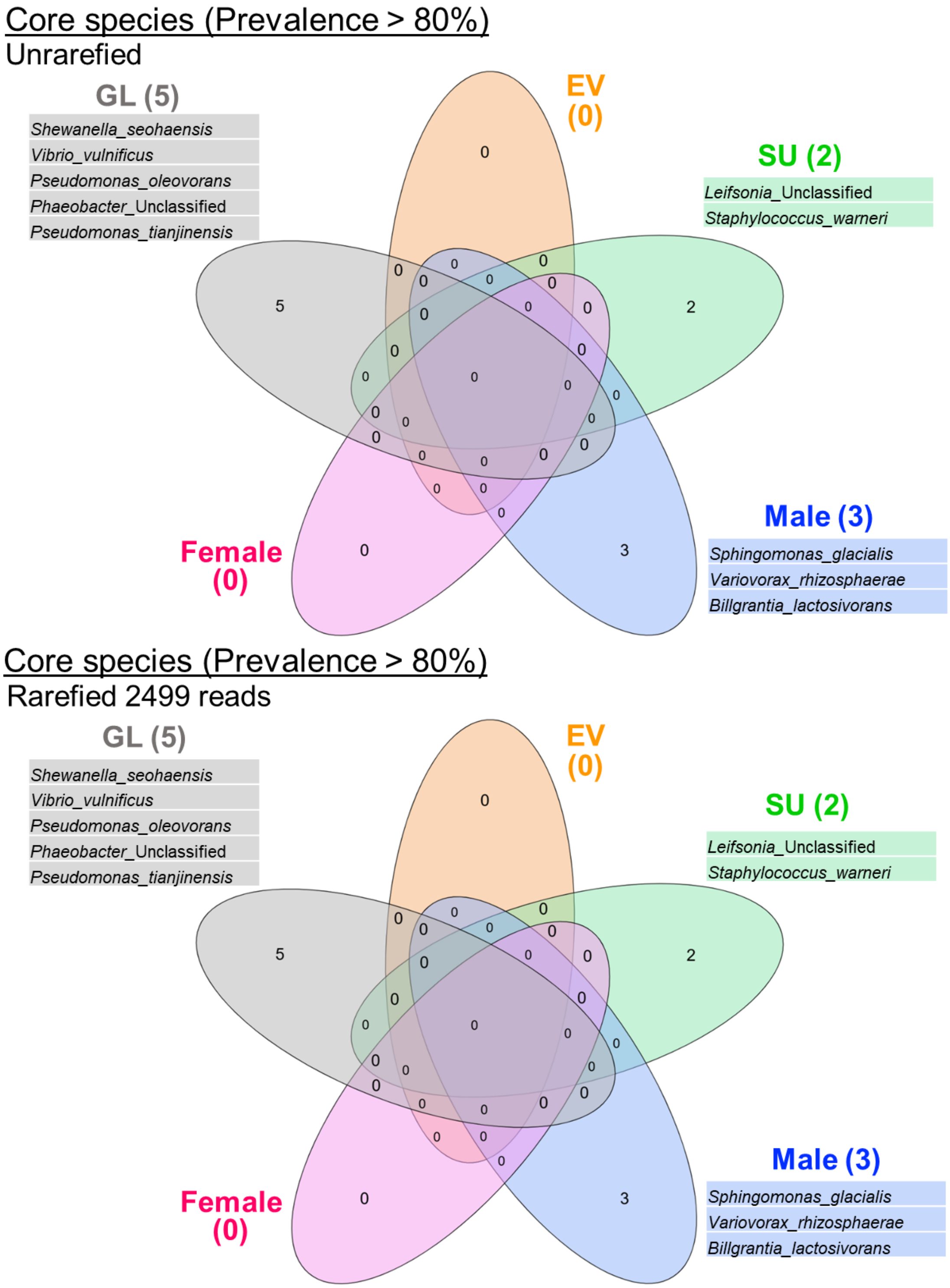

Supplemental Figure S10. Venn diagram analysis revealed no core species (prevalence >80%) shared across all developmental stages in either unrarefied or rarefied count tables. Core species identified within each developmental stage are listed in the corresponding Venn diagram.

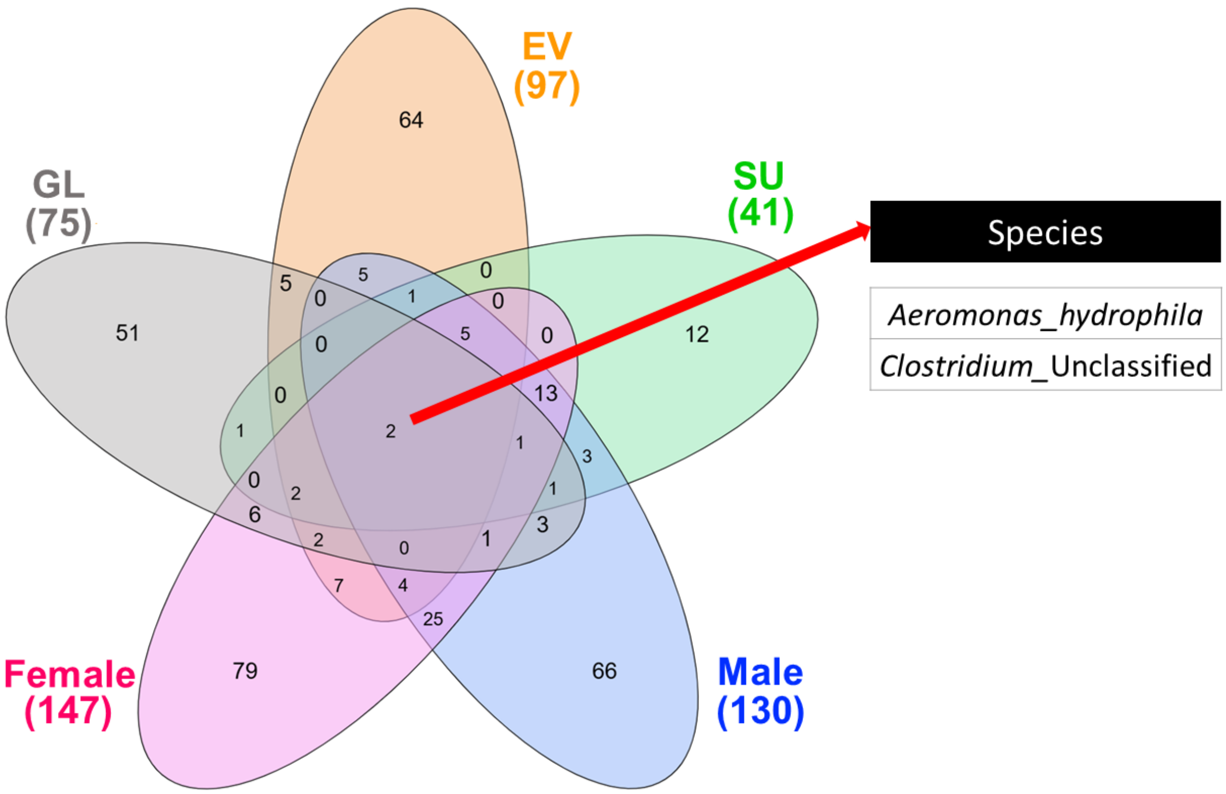

Supplemental Figure S11. Venn diagram analysis of gut microbiota identified two species (*Aeromonas hydrophila* and *Clostridium*_Unclassified) present across all developmental stages, with sex-determined adults showing higher bacterial richness (number of species) than younger eels.

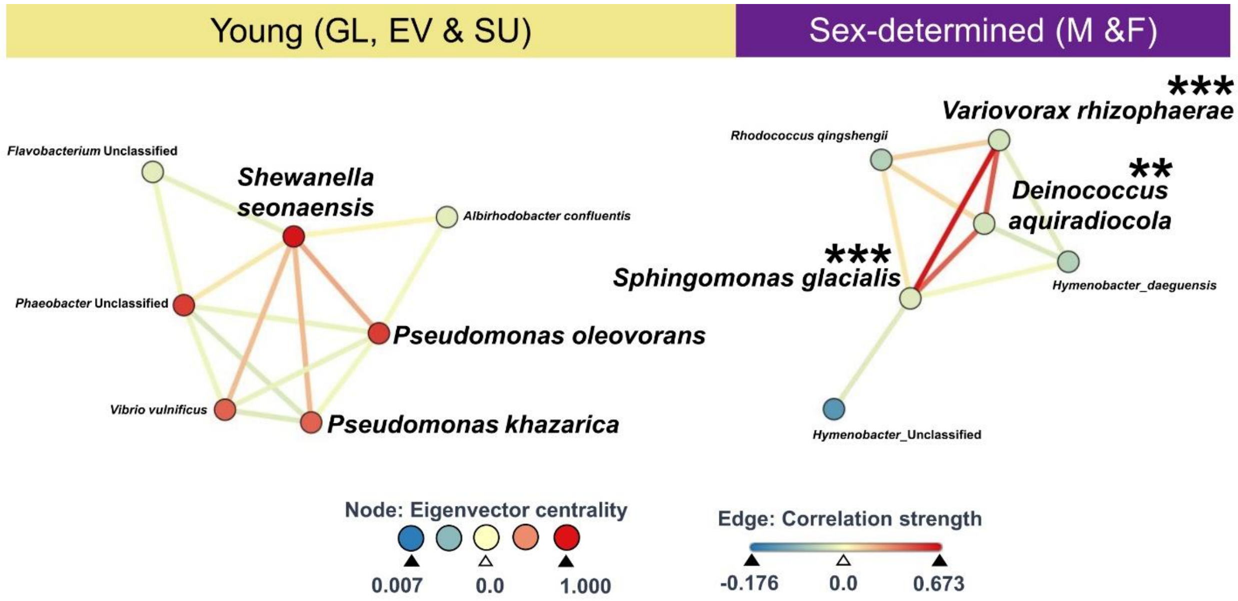

Supplemental Figure S12. Microbial co-occurrence subnetworks at the species level across eel life stages and correlations between microbial species and sex-specific gene expression. Each node represents a microbial species, with node color and size indicating the eigenvector centrality score. Edge color represents the correlation strength between nodes, reflecting associations between microbial species. Correlations were calculated using FastSpar with 20 iterations and 10,000 bootstrap replicates for *p*-value estimation. Asterisks indicate significance levels for correlations between microbial genera and the expression of *amh*: **p* < 0.05; ***p* < 0.01; ****p* < 0.001. Detailed correlation strength and p-values are available in **Supplemental Table S2.**

Supplemental Table S1. Correlation analysis between the abundance of bacterial genera and sex gene expressions (i.e., *amh* and *vtgr*). **p* < 0.05, ****p* < 0.001. Ranked by eigenvector centrality.

Developmental stage	Genus	Rank	*amh* (Correlation strength)	*vtgr* (Correlation strength)
Young	*Pseudomonas*	1	-0.177	-0.154
	Shewanella	2	-0.188	-0.119
	Vibrio	3	-0.143	0.054
	Phaeobacter	4	-0.120	-0.083
	Flavobacterium	7	-0.121	-0.076
	Albirhodobacter	9	-0.061	-0.113
	Acinetobacter	11	-0.099	-0.101
	Limosilactobacillus	12	0.012	-0.063
	Enterococcus	13	-0.090	0.090
	Cutibacterium	14	0.134	0.127
	Brevinemataceae_Unclassified	16	0.016	-0.049
	Chryseobacterium	16	0.004	-0.042
	Staphylococcus	19	-0.058	0.136
	[*Clostridium*]	20	-0.090	-0.050
	Leifsonia	23	-0.046	-0.093
Sex-determined	*Variovorax*	5	0.417 ***	-0.017
	Rhodococcus	6	0.126	0.040
	Sphingomonas	6	0.446 ***	-0.080
	Deinococcus	8	0.231 *	0.115
	Hymenobacter	10	0.142	-0.029
	Methylobacterium	10	0.032	-0.005
	Plesiomonas	15	0.048	-0.062
	Faecalimicrobium	21	0.002	0.057

Supplemental Table S2. Correlation analysis between the abundance of bacterial species and sex gene expressions (i.e., *amh* and *vtgr*). **p < 0.01, ***p < 0.001. Ranked by eigenvector centrality.

Developmental stage	Genus	Rank	*amh* (Correlation strength)	*vtgr* (Correlation strength)
Young	*Shewanella_seonaensis*	1	-0.16	-0.127
	*Phaeobacter*_Unclassified	2	-0.125	-0.114
	Pseudomonas_oleovorans	2	-0.118	-0.055
	*Flavobacterium*_Unclassified	3	-0.065	-0.05
	Vibrio_vulnificus	3	-0.157	-0.073
	Pseudomonas_khazarica	3	-0.051	-0.037
	Albirhodobacter_confluentis	4	-0.111	-0.042
Sex-determined	*Sphingomonas_glacialis*	5	0.447 ***	-0.094
	Variovorax_rhizophaerae	6	0.373 ***	-0.024
	Deinococcus_aquiradiocola	6	0.267 **	-0.082
	Hymenobacter_daeguensis	7	0.143	0.022
	Rhodococcus_qingshengii	7	0.187	0.024
	*Hymenobacter*_Unclassified	8	0.12	0.008
